# Stress Transfer Around Cross Passages in Shallow Tunnels: Effects of Volume Loss and Cross Passage Shape

**DOI:** 10.1007/s10706-026-03724-3

**Published:** 2026-05-19

**Authors:** Ahsan Saif, Munawar Hussain, Enrico Soranzo, Wei Wu

**Affiliations:** 1 Institut Für Geotechnik, University of Natural Resources and Life Sciences, Feistmantelstraße 4, 1180 Vienna, Austria; 2 Saudi Consulting Services, Sulaymaniyah, Riyadh, 12223 Kingdom of Saudi Arabia

**Keywords:** 3D finite element, Child tunnel, Cross passage, Hardening soil with small strain, Tunnel intersections, Underground construction

## Abstract

This study investigates the response of a parent tunnel (PT) lining to volume loss and cross-passage (CP) shape and how it affects the stress redistribution and deformation of the PT lining around the opening for shallow tunnels in cohesionless soils. Three-dimensional finite-element parametric analyses were carried out using the Hardening Soil with Small Strain constitutive model simulated varying soil densities, CP/PT size ratios (0.25–0.90), PT volume-loss values (0.25–1.0%) and four CP geometries (circular, square, modified horseshoe, inverted-D). Results show that increasing PT volume loss generally reduces residual hoop stresses at CP springlines and crown/invert while producing higher longitudinal stresses at the springlines. CP shape controls stress arching: circular openings concentrate hoop stresses at the springlines, whereas square, inverted-D and modified-horseshoe shapes transfer higher stresses to crown/invert regions. CP deformations decrease with increasing PT volume loss. Among the shapes considered, square openings exhibit the largest vertical deformations, while circular openings show the least. Normalised force–moment plots are developed to guide lining reinforcement around CPs and to inform shape-specific reinforcement detailing aimed at mitigating localised stress concentrations.

## Introduction

Modern transport and infrastructure tunnels often employ twin-tube or multi-tube layouts, in which two or more running tunnels (parent tunnels, PTs) are interconnected via cross-passages (CPs). These cross passages serve critical roles such as emergency egress, maintenance access, ventilation, and safety operations in case of incidents, among other uses, according to the guidelines of International Tunneling Association (ITA) Committee on Safety of Underground Facilities (COSUF), listed in Table 1. In essence, tunnel cross passages are integral components of modern underground infrastructure.

**Table 1:** Role of cross passages in tunneling infrastructure (ITA COSUF 2019)

During construction	During operation	During maintenance	During incidents
Housing of technical equipment/logistic for construction purposes	Protection of electrical, mechanical and environmental equipment	Provide appropriate working conditions	Means of escape providing an egress passage for escaping persons during self-rescue
Self-rescue and protection in the event of emergency incidents during construction	Management of cable passageways	Protection of the staff during train transit	Shelter area for escaping people and safety-relevant equipment until evacuation is complete
Assistance in tunnel ventilation during construction period	Assistance in tunnel air circulation and ventilation	Appropriate rest area during breaks and protection in case of relevant incidents	Access provision, logistic support and protection for emergency services

The spacing between cross passages and their dimensioning is typically determined by the regulations specific to each country. In accordance with ITA safety guidelines (ITA COSUF 2019), cross passages are commonly situated at intervals ranging from about 240 m to 500 m. The Chinese metro design code (GB 50157, 2013) recommends that the distance between two cross passages should not exceed 600 m. Guidelines from other countries outline the spacing of cross passages as 500 m (Austria), 800 m (France), and at least 750 m (Korea), 900 m (Germany) (Kim et al. 2007:08).

With cross passages, however, stress arching around the opening, introduces complex stress redistributions that must be taken into consideration for long term performance and durability of PT lining near CPs. Published research has identified that CP openings act as structural notches, altering load paths and leading to zones of concentrated stress, strain, deformation, or even damage in the lining near break-in or break-out interfaces (Yoo et al. 2019).

Similarly, studies in pressure-balance TBM tunnels show that CP construction can alter the loading regime of existing parent lining segments, particularly above and below CP openings (Epel et al. 2020). In a recent study, longitudinal cracks were observed in the thickening layer of a primary lining near CPs in Crossrail’s Bond Street SCL tunnels, back-analyses suggested that stress concentrations at the intersection crown played a major role (Su 2023).

Field observations and case studies have occasionally documented lining stress concentrations, deformations, or joint separation, adjacent to CP openings. However, these are not consistent, often lack detailed instrumentation, or fail to isolate the effects of CP geometry from other factors (groundwater, construction sequence, lining stiffness, etc.).

For example, Atzl et al. (2017) conducted field measurements to quantify the axial forces in the parent tunnel lining adjacent to cross passages in the Filder Tunnel using strain gauges in special monitoring segments. However, isolating the influence of the cross passage from other contributing factors, such as TBM thrust, annular grout pressure, groundwater, and the self-weight of the overburden, proved challenging and complex.

Atzl et al. (2019) performed detailed three-dimensional numerical analyses to assess the structural response CP openings in the Koralm Tunnel. Their findings indicated that CP construction in soft ground conditions was particularly demanding, and field measurements from Cross Passage 64 showed axial forces of approximately 5000 kN/m, while the implemented temporary bracing system effectively limited deformations to within the 6 mm.

However, the study does not provide a comprehensive assessment of the stress redistribution before and after the introduction of the CP opening, primarily due to the difficulty of isolating the influence of concurrent effects. This lack of guidance leads to tunneling practitioners often relying on ground freezing or extensive structural supports around CPs to mitigate stress concentrations near the opening (Frodl 2019). Both methods, however, contribute significantly to increased costs and might result in project delays.

Given the practical importance of CPs in tunnel safety, cost, and structural integrity, there is a strong engineering need to better understand how hoop and longitudinal stresses are redistributed near CP openings. In this paper, we present numerical results assessing the effect of volume loss in parent tunnel and the shape of cross passage on the stress redistribution near the CP openings in shallow tunnels built in cohesionless soils. The overarching goal is to provide validated insights that can refine guidelines for lining design around CP openings, reducing over-conservatism, improving safety, and optimising cost.

## Previous Studies

Three subjects form the conceptual and technical foundation of this study: (1) volume loss in tunnelling, (2) the effect of tunnel geometry on structural and ground stability, and (3) the design of tunnel cross-passages. The following sections summarise relevant theoretical developments and empirical findings from the literature. These sections are included to clarify the theoretical framework and to justify the modelling choices and design criteria employed in the subsequent analysis.

### Volume Loss of Parent Tunnel

Volume loss (sometimes referred to as “ground loss”) refers to the volume of ground lost (i.e., soil/rock that moves or is displaced) during excavation. It is often expressed as a percentage of the theoretical excavated volume, and it relates directly to ground/surface settlement and distortion around the tunnel (Ahmed and Iskander 2011).

Peck (1969) was the first to introduce the Gaussian distribution curve to model surface settlement troughs due to volume loss. Afterwards numerous studies were undertaken including empirical and analytical methods, and theoretical studies (Fang et al (2016), Franza and Marshall (2019), Strack (2002), Loganathan (2011), Klar and Klein (2014), Liu et al. (2020), Zhu et al. (2023), Lee et al. (1992).

Volume loss is often expressed as the relation between the area of the settlement trough versus the area of the tunnel. The volume loss parameter depends on the tunnel depth, the tunnel diameter, lining stiffness and the soil properties, it also has a key impact on the shape and extent of the settlement trough and therefore has a major influence on predicting the risk of damage to on ground infrastructure. Based on the study conducted by Möller and Vermeer (2008), the volume loss due to closed shield tunnelling in soft soil, can be composed of the following components:

- ground movements towards the face
- radial movements towards the shield
- ground movements towards the tail void
- radial movements of the lining segments
- long-term deformation of lining due to consolidation

Whereas in the case of conventional open face tunneling, the main sources of ground movements are:

- Deformation towards the unsupported tunnel face
- Radial ground movement towards the relatively ductile shotcrete lining

More recently Dimmock and Mair (2007) used the load factor approach to estimate the volume loss in London’s over consolidated clay relating to the volume loss at the tunnelling face ahead of open-face shields and ahead of a sprayed concrete lining. Netzel (2009) analysed settlement field data of three Dutch TBM-tunnelling projects excavated in soft soil in Netherlands and estimated the volume loss values for Dutch project in the range of 0.15% up to 1.5%.

Rezaei and Ahmadi-adli (2019) examined field monitoring data of surface settlements from Tabriz metro line excavated in sandy and fine grained alluvial. The obtained volume loss varied from 0.13 to 1.35%, with an average value of 0.46% for closed face tunnelling with TBM-EPB. Meanwhile, centrifuge tests were employed by Marshall et al. (2012) to examine the volume loss effect in addition to other parameters on greenfield soil displacements above tunnels in sandy ground through. They reported that trough width was shown to decrease with an increase of volume loss parameter.

Vu et al. (2016) conducted a theoretical study which aimed to estimate the volume loss when tunneling with limited C/D ratios in various soils with a focus on slurry shield tunneling. They reported that the volume loss increases when tunnelling with shallower overburden. Moreover, it is found that in the case of shallow tunnelling, the volume loss at the tunnel face has a major impact in total volume loss.

Zhang et al. (2017) compared numerical simulation results with field measurement data of two case studies to investigate the relationship between face support pressure and volume loss in soft soils. Zhao et al. (2019) reported several factors that may induce volume loss in the soil during mechanized tunneling process. Moreover, the authors investigated the relation between tunnel volume loss and the surface volume loss by using a 2D FE-model compared with empirical solutions.

Additionally, Kampas et al (2020) addressed the effect of volume loss due to the construction of a horse shoe shaped tunnel on the seismic behavior of tunnels through nonlinear finite element (FE) modelling. The results confirmed that the consideration of volume loss does not significantly affect the deformation field around the tunnel, however it does beneficially decrease the lining forces.

### Effect of Tunnel Shape

One of the factors that can have significant effect on the tunnel lining design is its shape. Though circular tunnels have the disadvantage of wasting a large part of the usable area particularly in the case of large tunnels, they also remain the most common type in tunnel construction due to their excellent stress arching properties and the stability they provide (Yingyongrattanakul et al. (2001), Yoon et al. (2014), Rostami et al. (2016).

However, more often tunnels may be required to be constructed with different shapes depending on their function, ease of construction, spacing requirements, intended use and the nature of the surrounding geological medium. Several researchers have conducted investigations on the response of tunnel lining according to different shapes for instance.

González-Nicieza et al. (2008) investigated the effect of the tunnel shape on the determination of radial displacements using a modified convergence confinement method (CCM). Three typical tunnel cross-section shapes were considered for the three-dimensional analysis. The results showed the significant effect of tunnel shape on the overall stability and hence the need to consider the shape of the tunnel cross-section in the CCM. Palassi et al. (2008) compared analytical and numerical analysis to estimate the bending moment and axial forces developed in the lining of tunnels with circular cross section.

In the same context, Abdellah et al. (2018) investigated the effect of three excavation shapes (circular, square and horseshoe) on the stability of underground shallow tunnels excavated in limestone and rock mass using series of 2D FE models employing the Mohr–Coulomb (MC) failure criterion with and without joint inclusion. Their findings indicated that the weak performance of a tunnel opening occurred with a square-shaped opening especially when rock joints are present. In addition, failure occurred in of “cone-shaped” form around flat or right-angled surfaces (e.g. in square tunnels and the invert of horseshoe tunnels).

Patil et al. (2018) proposed to investigate the performance of a square with rounded corners under seismic loading conditions by using FE analysis. They observed that the unconventional shape of the square tunnel with rounded corners showed lower bending moments when compared with conventional square tunnel under seismic loading. Moreover, the authors concluded that the circular tunnel performs better than other tunnel shapes under seismic loading.

Tien et al. (2020) employed an improved Hyperstatic Reaction Method (HRM) which was compared with the results of numerical models. Three tunnel shapes (circular, sub-rectangular and horseshoe) were investigated using the same tunnel clearances and geotechnical conditions. The findings confirmed that the circular tunnel shape is the best one in terms of tunnel lining stability. Moreover, the authors reported that the tunnel shape has a relatively strong influence on the bending moments than on the normal forces in the similar geotechnical conditions.

In the study presented by Huang et al. (2020), numerical simulation was adopted to explore the influence of different tunnel shapes of the same cross-section on the transient pressure variation induced by the passage of the same train through the tunnels at the same speed. The authors stated that a circular tunnel with a smaller cross-sectional perimeter is recommended for engineering applications, as not only is the pressure lowest, but also the airflow ahead propagates in a spherical form in this type of tunnel.

Manasa and Maji (2024) used two-dimensional numerical analyses to study the effect of tunnel shape in compressible rocks, and once again it was concluded that the deformation of the circular shape was less than that of the horseshoe shape and inverted-D tunnel, in addition the radius of the plastic zone was less for the circular and horseshoe shaped tunnels compared to the inverted-D shape. As stress concentration is greater on the sharp edges where the radius of curvature is greater, the inverted-D tunnel and the horseshoe tunnel show greater deformations, particularly at the invert, compared to the circular tunnel.

A recent study by Kota et al. (2024) examined the dynamic response of three tunnel shapes (rectangular, arched-back, horse-shoe) in a hypothetical tunnel intersection within blocky rock mass conditions. They employed the 3D distinct-element code, 3DEC, for numerical modeling of earthquake-induced displacements. The analysis of total displacements revealed that block detachment occurred mainly in the vicinity of the tunnel's crown region, particularly at the intersection point. In addition, square-shaped tunnels showed the highest block displacements, followed by arched and horseshoe-shaped tunnels.

Using trapdoor experiments, Zhang et al. (2024) studied the effect of existing tunnels on the evolution of the soil arching, by varying the shape of the tunnel (circular and rectangular). The results showed that the stress redistribution around the rectangular tunnel was significantly greater than that of the circular tunnel of the same size. However, circular tunnels had systematically higher maximum surface displacements than rectangular tunnels.

### Cross Passages in Twin Tunnels

Relatively few investigations provide analytical solutions for lateral openings in tunnels. Among these, the Kirsch model offers one of the earliest attempts to derive an analytical approach describing the stress redistribution around a circular opening in an infinite elastic plate under plane-stress conditions, (Hoek and Brown (1980)).

This model idealizes the parent tunnel as an unwrapped infinite plate subjected to uniform loading and provides an approximate estimation of radial and tangential stress redistribution around the opening. Although its assumptions are conservative and limited to circular openings, the Kirsch model remains widely used due to its simplicity. Other analytical approaches also exist: Roark’s formulas (Young 1989) extend solutions to elliptical openings, while Peterson (Pilkey and Pilkey 2008) provides stress concentration factors for circular openings in pressurized pipes.

**Fig. 1:**
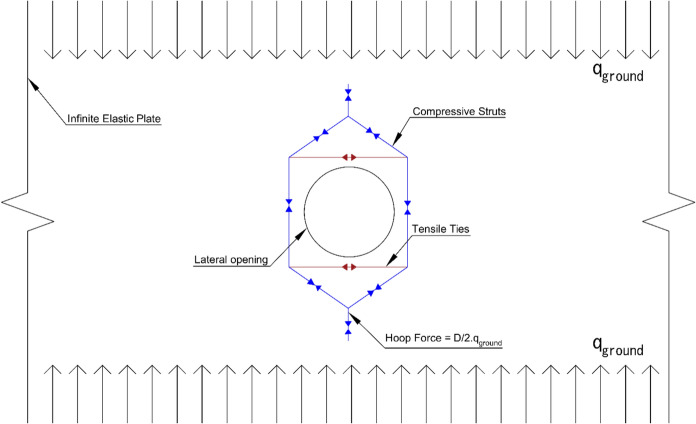
Strut and tie model (adapted from Spyridis and Bergmeister (2015))

Another analytical solution was provided by Bergmeister et al. (1993) for openings in a two-dimensional projection, known as the strut-and-tie model. In this approach, the compressive resultant is derived from the hoop compressive forces in the parent tunnel and directed toward the vertical centreline of the opening. That resultant is then decomposed into two inclined compressive struts, which are transmitted into the sides of the opening, and one tensile tie at the crown/invert of the opening, forming a force equilibrium above and below the opening as shown in Fig. 1.

As a result, compressive forces are transferred to both sides of the opening. The components of the strut and tie model are proportional to the original (parent tunnel) hoop force with the magnitude determined by the size of the parent tunnel and the geometry of the cross passage opening.

Most studies undertaken to investigate the effect of CP construction on the parent tunnel mostly comprises numerical studies using three-dimensional FE or finite difference (FD) analyses. Most of these studies are either case specific studies or generalized parametric studies. For instance, Tsuchiyama et al. (1988) conducted elastic numerical analyses to assess the influence zone around a parent tunnel during the construction of an access tunnel.

Pottler (1992) examined a representative tunnel junction configuration within the English Channel Tunnel Project to assess the need for increased support lining thickness near the intersection area. Swoboda et al. (1998) used finite element modelling to design and evaluate the stability of an intersection between the parent tunnel and an escape tunnel in the Schonberg Tunnel Project in Austria.

Hsiao et al. (2005) and Sjoberg et al. (2006) employed numerical analyses to investigate the behavior of tunnel intersection areas in the Hsuehshan Tunnel in Taiwan and the Citybanan Tunnel in Stockholm, respectively. Notably, Hsiao et al. (2005) delved into the deformational response of tunnel junctions and recommended reinforcement for specific intersection areas using a combination of numerical analyses and artificial neural networks. Forder et al. (2008) and Schikora et al. (2013) presented relevant studies showcasing the potential of optimized design using 3D numerical modelling for tunnel junctions.

More recently parametric studies were conducted to better understand the influence of a CP on its PT. Spyridis and Bergmeister (2015) parametrically investigated the effect of a perpendicular lateral breakout on the structural response of a shallow running tunnel in a three-dimensional FE framework. The authors concluded lateral breakouts cause significant longitudinal tension in the crown-invert area, high compression in the hoop direction on the springlines in the running tunnel and the extent of stress distribution extends to about 1 diameter of the breakout opening.

In a similar study conducted by Ke et al. (2019), a 3D FD sensitivity analysis was carried out to assess the impact of various factors on tunnel cross passages. The study yielded findings similar to previous research; however, a particularly interesting aspect of the study was to investigate the effect of the angle at which the cross passage intersects the parent tunnel. The results revealed that at intersection angles between 75 and 90°, the cross-passage’s stress distribution remained relatively stable.

Saif et al. (2025) performed FE parametric analyses and reported that compressive hoop stresses increase significantly around the springlines of CP, with largest values typically occurring at a CP/PT ratio of 0.5. While tensile longitudinal stresses at the crown/invert of the CP peak at CP/PT ratio of 0.5 to 0.75. In another study, centrifuge experiments also showed interesting preliminary results (Saif et al. 2024).

### Conclusion

From a review of literature, it can be assessed that the effect of shape of parent tunnel and the volume loss resulting from its excavation are key factors affecting the induced ground deformations, tunnel lining stresses and lining design. However, none of the previous works provides an understanding of how PT volume loss and CP shape may affect the design of the cross passages. The current study aims to explore this by conducting a series of 3DFE analyses, investigating the effect of volume loss resulting from PT excavation and CP shape on the stress regime around the CP opening.

## Research Methodology

**Fig. 2:**
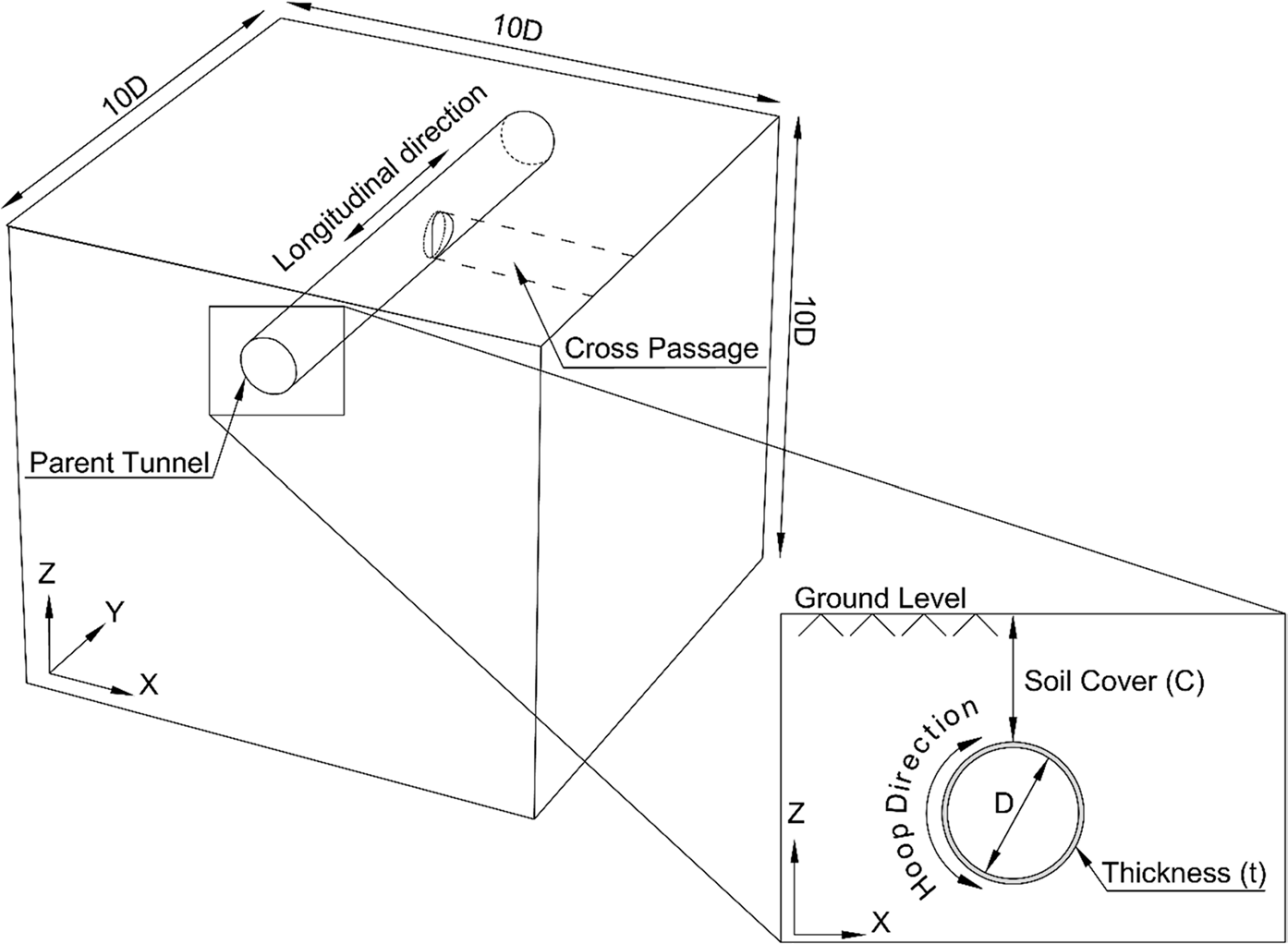
3D-FE model illustrating the parent tunnel and cross passage

A 3D FE approach was employed to investigate stress redistribution due to CP openings on the PT lining using PLAXIS 3D. The numerical model spanned the diameter of the parent tunnel ten times in all three directions, as depicted in Fig. 2, to minimize boundary interference.

The model comprised approximately 125,000 to 150,000 10-noded tetrahedral volume elements and 250,000 to 280,000 nodes, depending upon the size/shape of the CP opening. The mesh size was refined near the CP opening (1 CP diameter longitudinally on each side), so that each mesh element was equal to or smaller than the thickness of the lining (400 mm). The model was unconfined at the top, normally restrained at the sides, and fully fixed at the bottom.

A total of 72 analyses were carried out on varying soil strength, CP/PT ratio and volume loss percentages, as listed in Table 2. The results obtained through these three-dimensional parametric analyses can be an excellent resource for comparison with experimental or field data to further our understanding of stresses in parent tunnel linings around cross passage openings. The following procedure was followed to analyze the numerical model and extract force-moment data.

- *Stage* 1: Initial geostatic stress state was established based on gravity.
- *Stage* 2: Excavation of soil and complete installation of parent tunnel lining with the radial contraction applied to model the volume loss.
- *Stage* 3: Introduction of the cross-passage opening in the parent tunnel. Only the CP opening was simulated, and further soil excavation and lining installation in the cross passage was not carried out. Only the opening is modelled, as the initial introduction of the CP opening without any support is the worst-case scenario for the PT lining, generating the largest stresses in the lining.
- Extraction of force-moment values (hoop and longitudinal) along the cross sections (springlines and crown/invert). These force and moment values were normalised with the average hoop force and hoop moment respectively at the springlines of the PT before CP breakout.
- Plotting of the normalised values, closest to the cross passage (at springlines and crown/invert) against CP/PT ratio.

**Table 2:** List of analyses conducted

Analysis No	Volume loss, *V*_s_	Soil relative density, *D*_*p*r_	Size ratio*, CP/PT*
*CP: Circular*			
1	0.25%	20	0.25
2			0.50
3			0.75
4			0.90
5		50	0.25
6			0.50
7			0.75
8			0.90
9		100	0.25
10			0.50
11			0.75
12			0.90
13	0.50%	20	0.25
14			0.50
15			0.75
16			0.90
17		50	0.25
18			0.50
19			0.75
20			0.90
21		100	0.25
22			0.50
23			0.75
24			0.90
25	1.00%	20	0.25
26			0.50
27			0.75
28			0.90
29		50	0.25
30			0.50
31			0.75
32			0.90
33		100	0.25
34			0.50
35			0.75
36			0.90
*CP: Square*			
37	0.25%	50	0.25
38			0.50
39			0.75
40			0.90
41	0.50%		0.25
42			0.50
43			0.75
44			0.90
45	1.00%		0.25
46			0.50
47			0.75
48			0.90
*CP: Modified horseshoe*			
49	0.25%	50	0.25
50			0.50
51			0.75
52			0.90
53	0.50%		0.25
54			0.50
55			0.75
56			0.90
57	1.00%		0.25
58			0.50
59			0.75
60			0.90
*CP: Inverted-D*			
61	0.25%	50	0.25
62			0.50
63			0.75
64			0.90
65	0.50%		0.25
66			0.50
67			0.75
68			0.90
69	1.00%		0.25
70			0.50
71			0.75
72			0.90

### Types of Analyses Conducted

Two sets of parametric analyses were conducted. The first type modelled the effect of parent tunnel volume loss on the stress redistribution around a circular cross passage. CP/PT ratios of 0.25 to 0.90 were analysed at varying initial parent tunnel volume losses of 0.25%, 0.5% and 1.0%. These PT volume loss values represented different construction rates at which the parent tunnel can be excavated and the response of the surrounding strata to the excavation.

The influence of soil strength was incorporated by modelling two different relative densities (*D*_pr_) of 25 and 100% for the surrounding granular soil. These values were used in order to capture the behaviour of granular soils under loose (*D*_pr_ = 25%) and dense (*D*_pr_ = 100%) conditions, respectively, and allow for a comparative assessment of the ground-lining interaction under different soil stiffnesses.

**Fig. 3:**
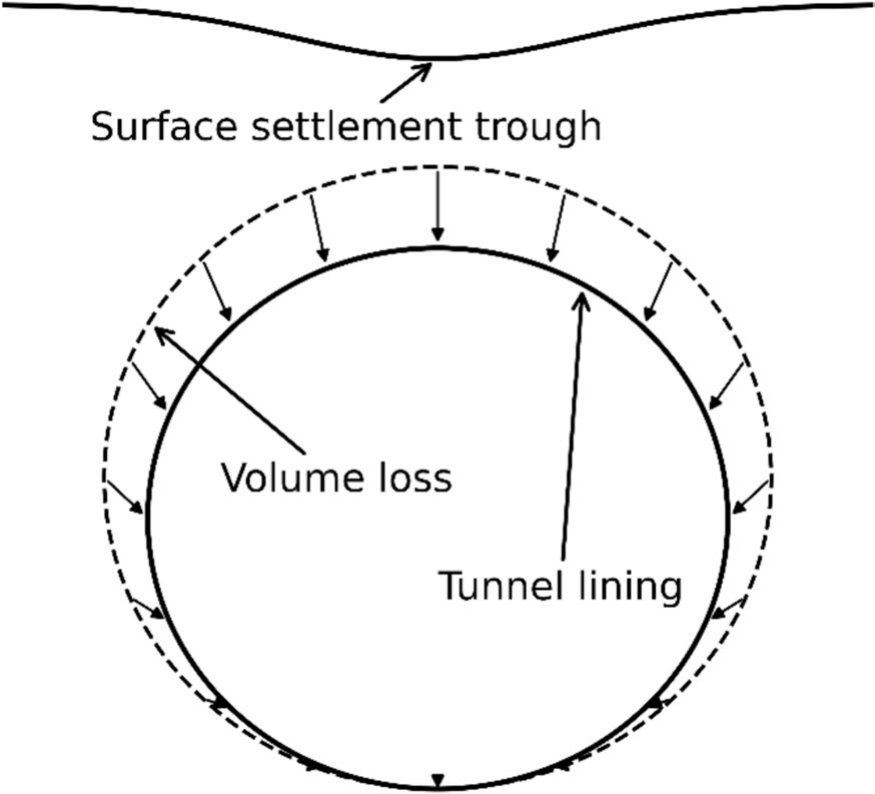
Schematic representation of the modelled volume loss around the parent tunnel

Tunnelling-induced volume loss was represented via the surface contraction method. A prescribed negative displacement (radial contraction) was applied normal to the excavation boundary, such that the total reduction in the tunnel excavated area corresponds to the target volume loss percentage, as illustrated in Fig. 3. This contraction is implemented as an initial strain (ε₀) on the surface mesh, after which the FE solver computes the resulting stress redistribution and ground surface settlements as the tunnel boundary contracts.

This contraction does not directly generate forces in the lining; instead, it induces deformations in the surrounding soil at the surface as the it converges towards the excavation boundary and a redistribution of stresses. As the tunnel boundary contracts, the soil moves inward, leading to stress relief near the excavation and the development of stress arching around the tunnel. This redistribution alters the load transfer mechanism between the ground and the lining, typically reducing the total loads attracted towards the tunnel lining.

The radial contraction method was adopted instead of excavation-based modelling for the 72 analyses, as it significantly reduces analysis time and storage requirements compared to modelling step-by-step excavation for a 100 m tunnel length. To assess the effect of cross passage geometry on the structural response of the parent tunnel lining, a separate set of analyses was conducted for four distinct CP shapes: circular, square, modified horseshoe, and inverted-D.

These shape effect analyses were performed using a uniform soil relative density of 50% and the volume loss of the PT corresponded to 0.25%, 0.50% and 1.0%. Furthermore, the geometry of each cross passage was modelled to maintain approximately equal excavation areas for each CP/PT size ratio, thereby isolating the effect of shape from that of excavation magnitude.

**Fig. 4:**
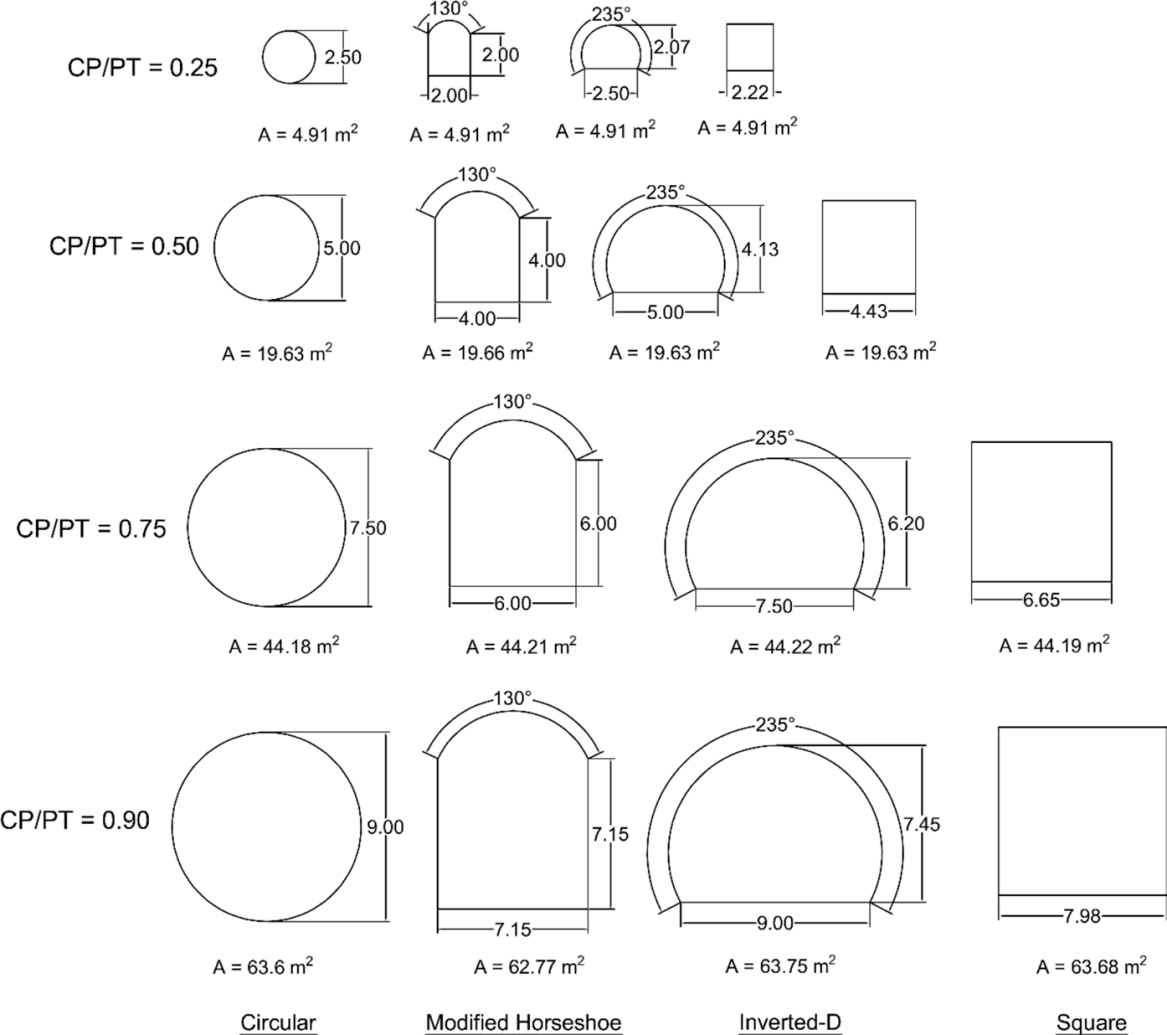
CP shapes corresponding to different CP/PT ratios

The geometric configurations of the cross-passage shapes are illustrated in Fig. 4.

### Soil and Tunnel Properties

The soil was modeled as a non-cohesive granular material using the non-linear elasto-plastic constitutive model Hardening Soil with Small Strain Stiffness (HSS) (Benz 2007;Vermeer 1978; Schanz et al. 1999), while the tunnel lining was modelled as a linear elastic plate element. The required HSS model parameters are typical values for cohesionless soils and were determined using Eqs. 1 – 11 (Brinkgreve et al. 2010). The parent tunnel had a diameter of 10 m, a length of 100 m, with a lining thickness of 400 mm and Young’s modulus of 30 GPa. Properties for the soil and lining are listed in Tables 3 and 4 respectively.

**Table 3:** Soil properties

Parameter	*D*_pr_ = 20%	*D*_pr_ = 50%	*D*_pr_ = 100%
*γ*_unsat_ (kN/m^3^)	15.8	17.0	19.0
*γ*_sat_ (kN/m^3^)	19.32	19.80	20.60
*E*_50_^ref^ (kPa)	12,000	30,000	60,000
*E*_oed_^ref^ (kPa)	12,000	30,000	60,000
*E*_ur_^ref^ (kPa)	36,000	90,000	180,000
*G*_o_^ref^ (kPa)	73,600	94,000	128,000
m (-)	0.637	0.543	0.387
*γ*_0.7_ (-)	0.00018	0.00015	0.00010
ϕ' (°)	30.50	34.25	40.50
Ψ (°)	0.50	4.25	10.50
K_0_	0.492	0.437	0.350
*R*_f_ (-)	0.975	0.938	0.875

**Table 4:** Tunnel lining properties

Parameter	Value
Unit Weight, γ (kN/m^3^)	25
Secant modulus, E (GPa)	30
Poisson ratio, μ (-)	0.15
Thickness, t (mm)	400

1 $${\gamma}_{unsat}\left[\frac{kN}{{m}^{3}}\right]=15+ 4.0\cdot \frac{D\mathrm{r}}{100}$$

2 $${\gamma}_{sat}\left[\frac{kN}{{m}^{3}}\right]=19+ 1.6\cdot \frac{D\mathrm{r}}{100}$$

3 $${E}_{50}^{ref}\left[kPa\right]=60000\cdot \frac{D\mathrm{r}}{100}$$

4 $${E}_{oed}^{ref}\left[kPa\right]=60000\cdot \frac{D\mathrm{r}}{100}$$

5 $${E}_{ur}^{ref}\left[kPa\right]=180000\cdot \frac{D\mathrm{r}}{100}$$

6 $${G}_{0}^{ref}\left[kPa\right]=60000+68000\cdot \frac{D\mathrm{r}}{100}$$

7 $$m\left[-\right]=0.7- \frac{D\mathrm{r}}{320}$$

8 $${\gamma}_{0.7}\left[-\right]=(2- \frac{D\mathrm{r}}{100})\cdot {10}^{-4}$$

9 $${\varphi }{\prime}\left[^\circ \right]=28+ 12.5\cdot \frac{D\mathrm{r}}{100}$$

10 $$\psi \left[^\circ \right]=-2+ 12.5\cdot \frac{D\mathrm{r}}{100}$$

11 $${R}_{f}\left[-\right]=1- \frac{D\mathrm{r}}{800}$$

### Data Extraction from Numerical Models

**Fig. 5:**
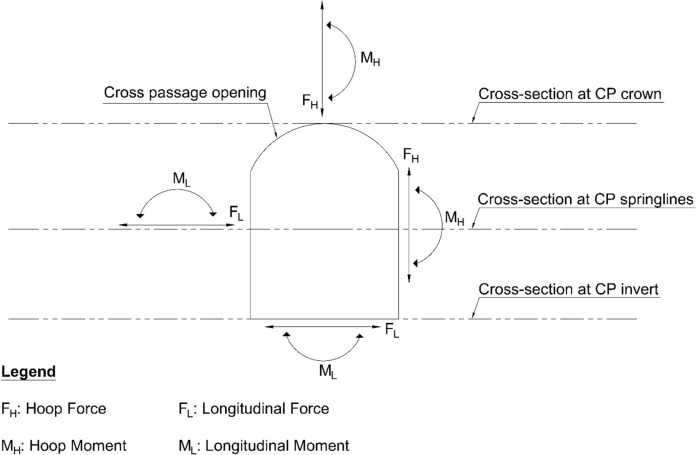
Forces/moments extracted along the illustrated cross-sections (modified horseshoe section shown as an example)

Results from 3D FE models were extracted in the form of forces and moments within the PT lining, both in the hoop and longitudinal directions, before and after the introduction of the CP opening. Two cross sections, one at the springlines level and another at the crown height of the CP, were cut throughout the length of the parent tunnel, along which the force and moment values were extracted. For modified horseshoe and inverted-D shapes, forces/moments along a cross section at the invert were also extracted as shown in Fig. 5.

**Fig. 6:**
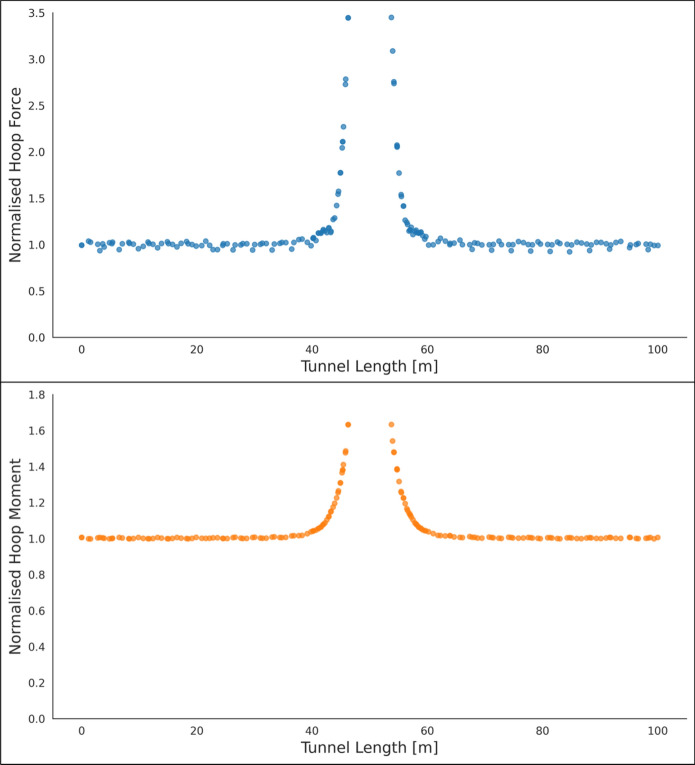
Normalised hoop force and moment along the parent tunnel length at springlines of the CP (*CP/PT* = 0.75, Soil *E*_50_.^ref^ = 60 MPa, *C/D* = 1, *V*_S_ = 0.25%)

**Fig. 7:**
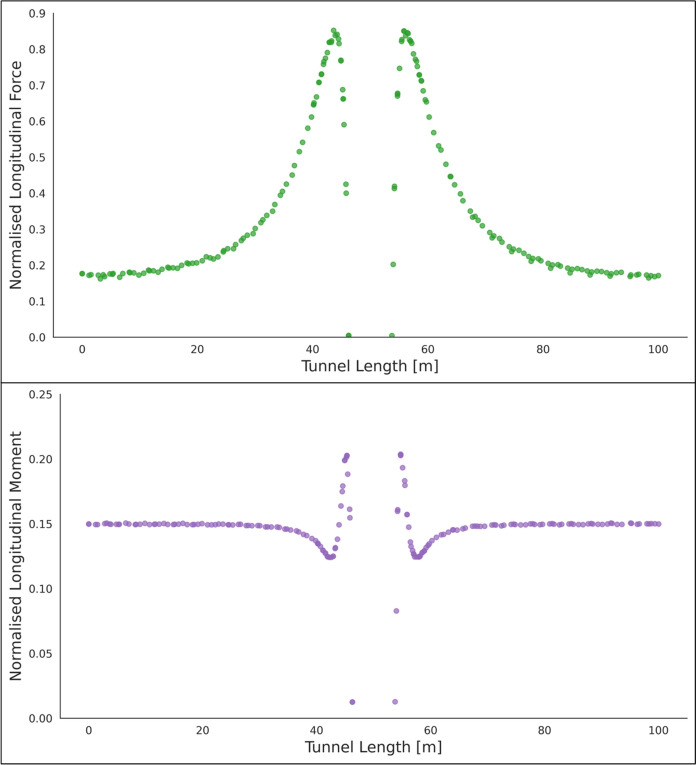
Normalised longitudinal force and moment along the parent tunnel length at springlines of the CP (*CP/PT* = 0.75, Soil *E*_50_.^ref^ = 60 MPa, *C/D* = 1, *V*_S_ = 0.25%)

**Fig. 8:**
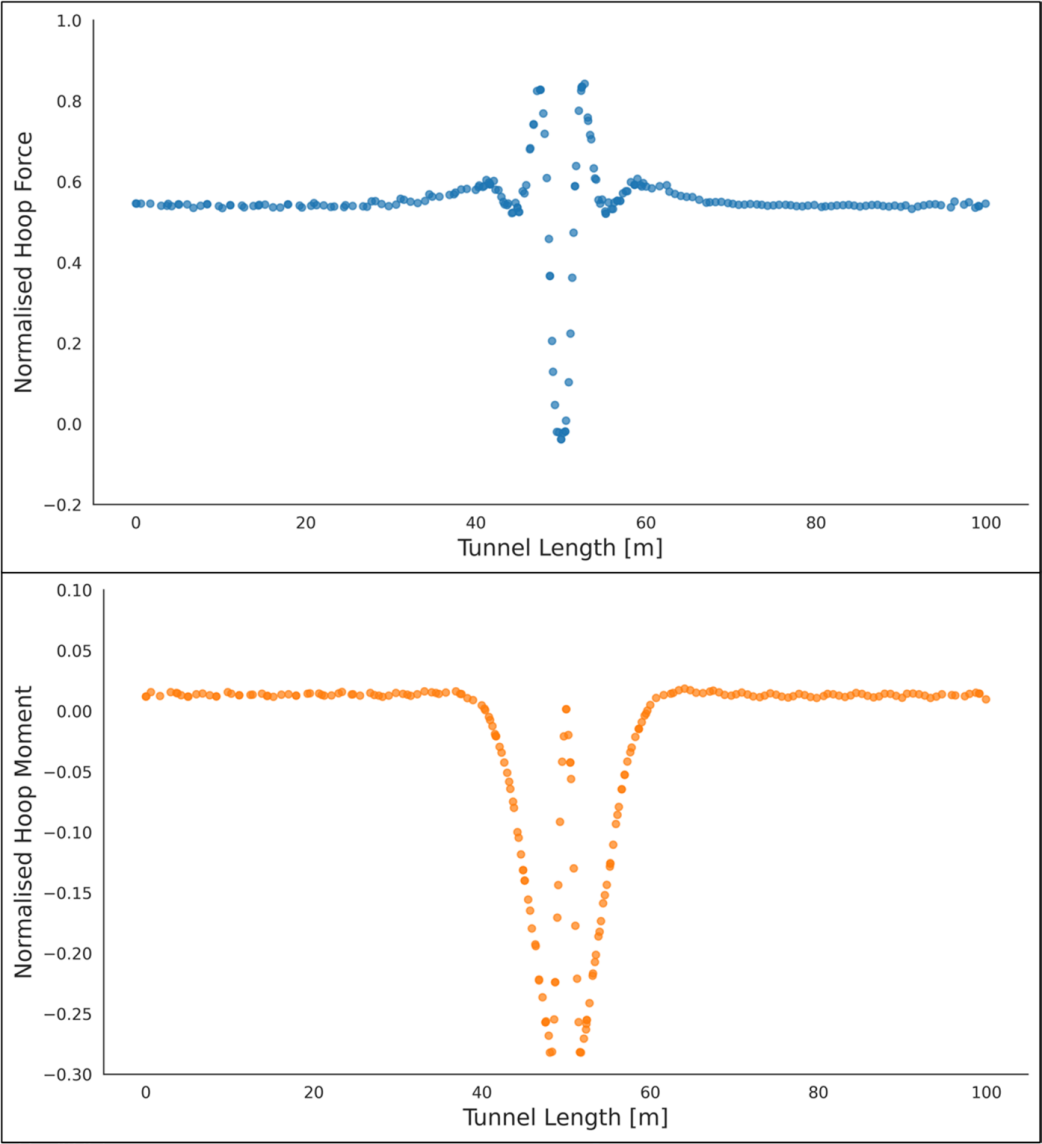
Normalised hoop force and moment along the parent tunnel length at crown of the CP (*CP/PT* = 0.75, Soil *E*_50_.^ref^ = 60 MPa, *C/D* = 1, *V*_S_ = 0.25%)

**Fig. 9:**
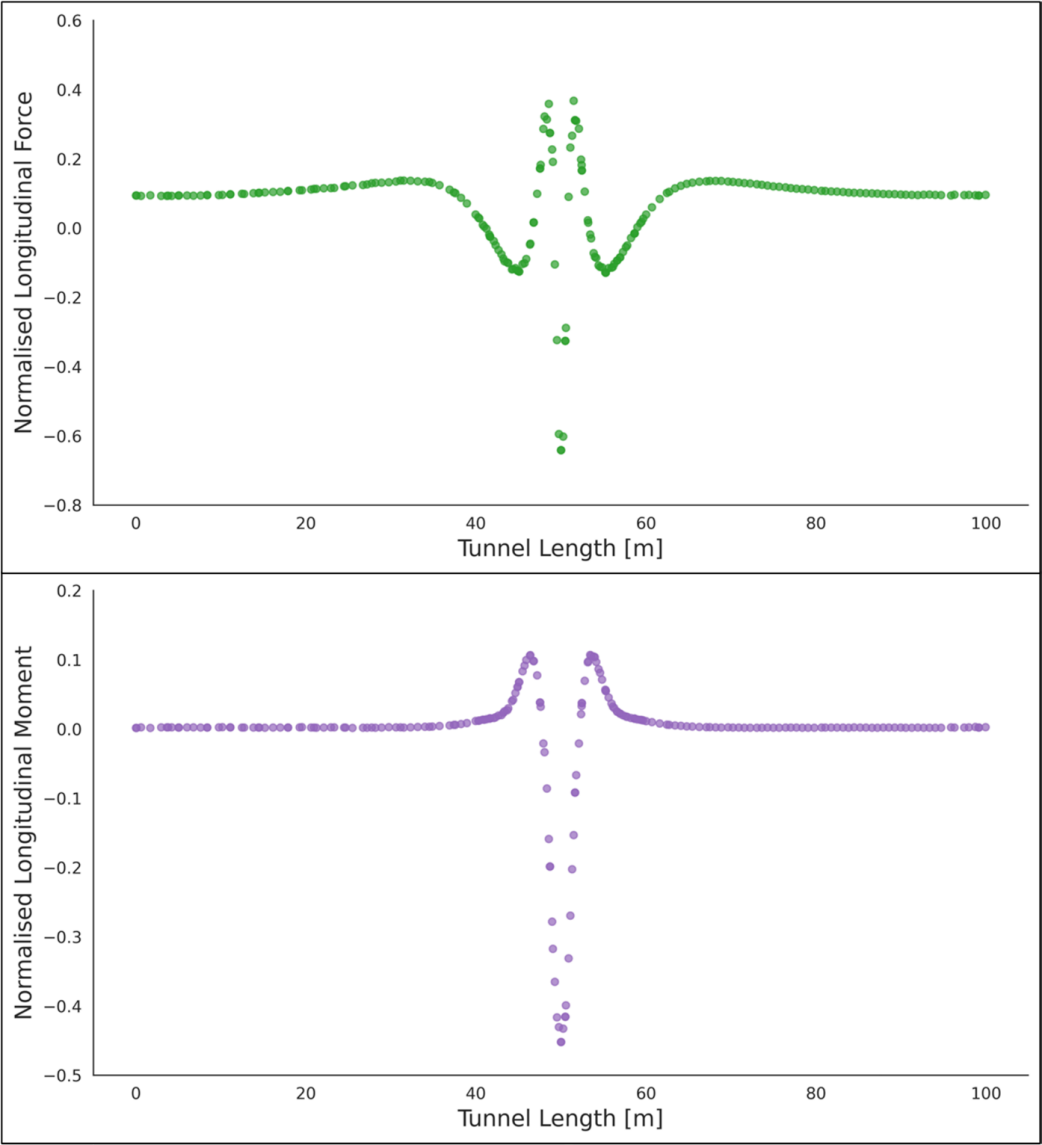
Normalised longitudinal force and moment along the parent tunnel length at crown of the CP (*CP/PT* = 0.75, soil *E*_50_.^ref^ = 60 MPa, *C/D* = 1, *V*_S_ = 0.25%)

The forces (hoop and longitudinal) along both cross sections were then normalized with average hoop force at the springlines of PT prior to CP opening, while moment values (hoop and longitudinal) were normalized with average hoop moment at the springlines of PT before CP opening. Sample plots of these normalized force and moment distributions along the cross sections and in both directions (hoop and longitudinal) are shown in Figs. 6, 7, 8 and 9. Lastly, the largest of these normalized values, closest to the CP opening, were plotted against CP/PT ratio.

The reason for utilizing the average hoop force and moment at the springlines of the parent tunnel to normalize all results is that these values are easily obtainable from 2D plane-strain analyses as well. Therefore, the objective is to develop internal forces plots that can be used in conjunction with simplified 2D analysis to determine the stress regime around the CP opening. Subsequently, the derived forces and moments can be employed to design the strength, thickness, and steel reinforcement of the tunnel lining around the CP.

## Results and Discussion

### Model Calibration

The 3D FE numerical model was validated by comparison with analytical closed-form solutions and with an equivalent 2D model before conducting parametric study. Forces within the parent tunnel lining were assessed using the 3D model (prior to the CP opening) and compared with the 2D model and analytical methods. The analytical solutions employed to evaluate the forces and moments within the tunnel lining included:

- Ranken, Ghaboussi, & Hendron (1978)
- Einstein and Schwartz (1979)
- Curtis and Muir Wood (1975–6)

These analytical solutions typically assume plane strain conditions in an isotropic, homogeneous, elastic medium with an elastic lining, wished into place and applicable only to circular tunnels. Further details related to the analytical models can be found in their respective publications.

Since the analytical solutions consider wished in place conditions, the 2D and 3D numerical models also employed a wished-in-place circular parent tunnel with an elastic lining, and radial contraction was not applied on the lining to model wished in place conditions. For simplicity, a C/D ratio of 2 and a relative density (*D*_r_) of 100% were used for this validation process. The soil and lining properties for the analytical solutions were consistent with those listed in Tables 3 and 4.

**Fig. 10:**
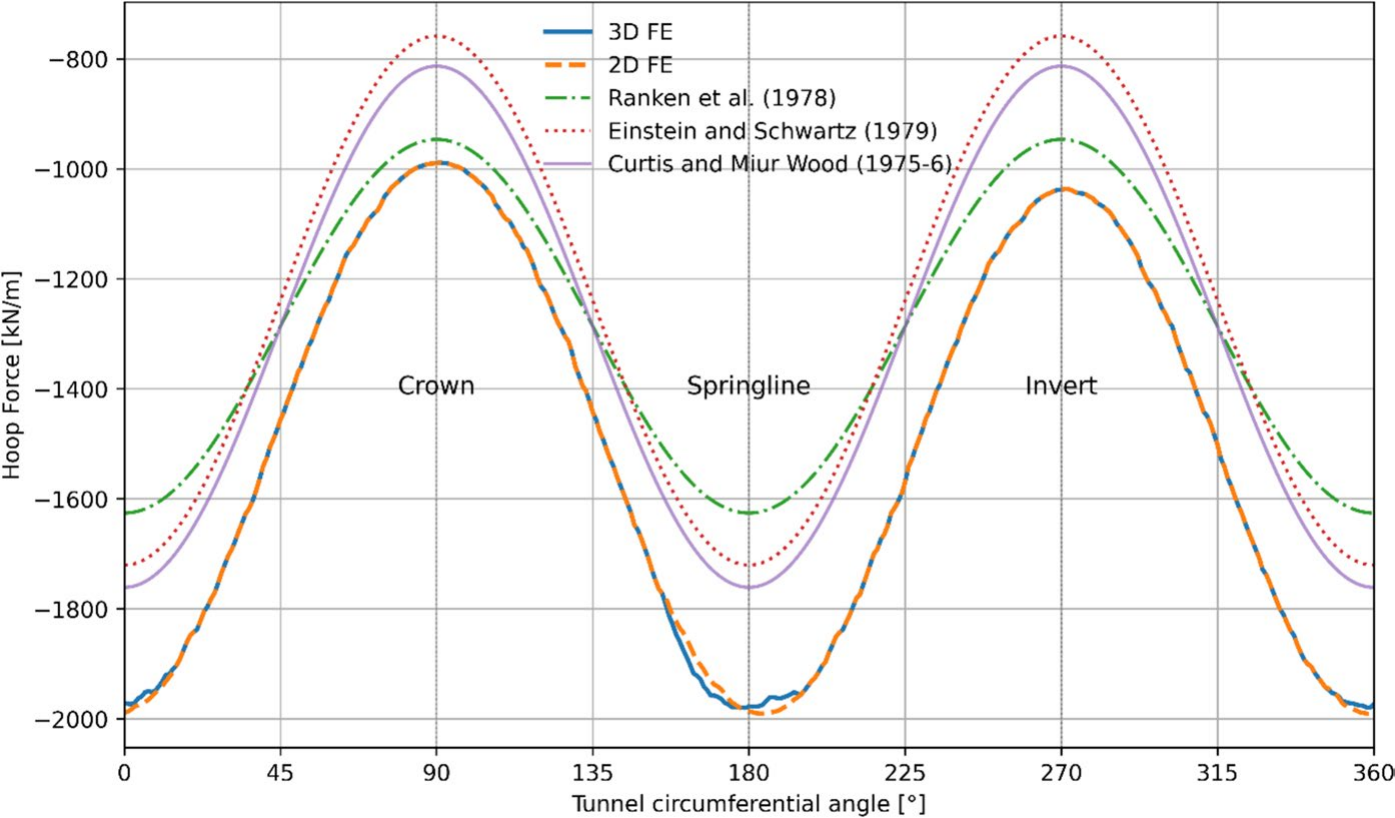
Hoop force within the PT lining obtained from analytical, 2D and 3D methods

**Fig. 11:**
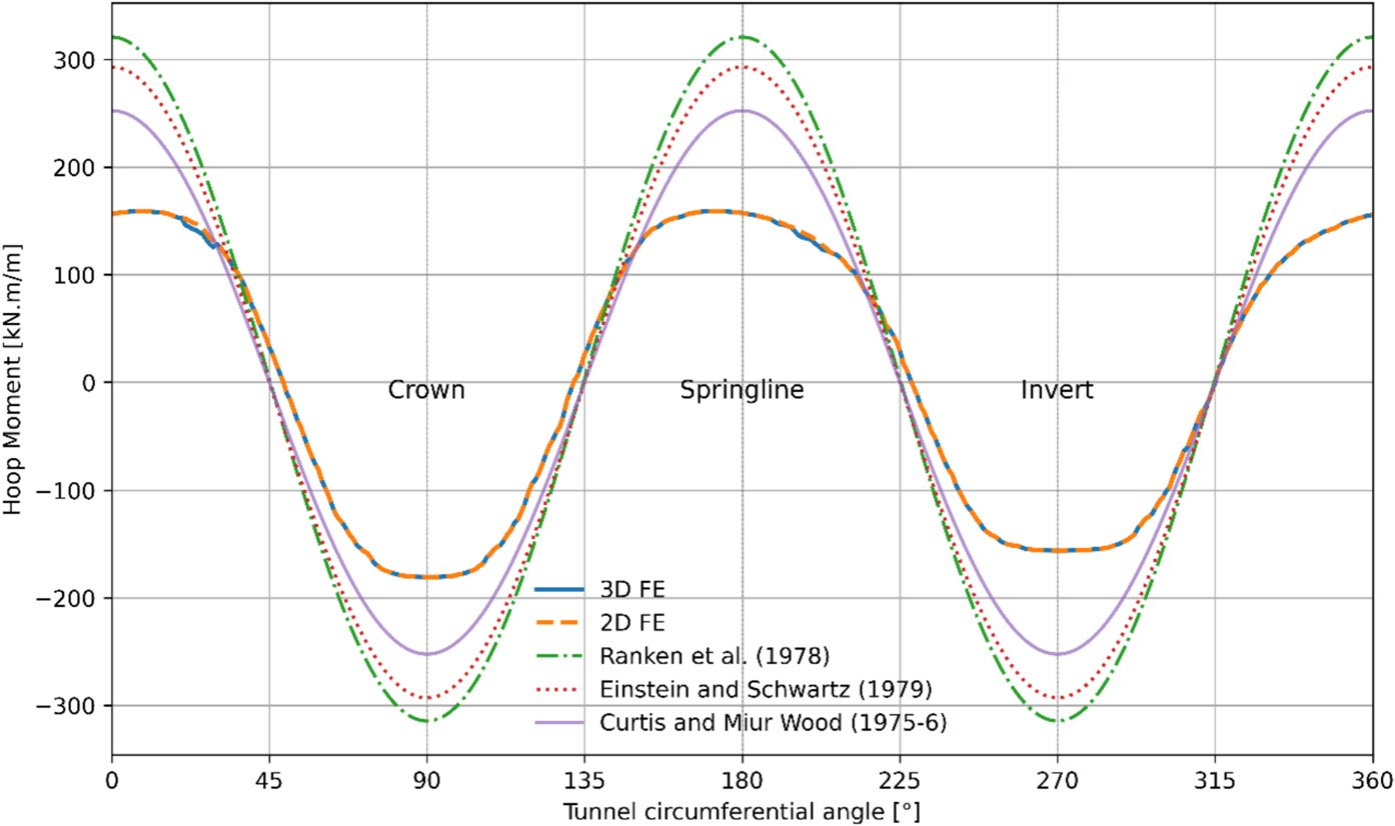
Hoop moment within the PT lining obtained from analytical, 2D and 3D methods

As shown in Figs. 10 and 11, the forces and moments within the lining derived from the analytical solutions closely match the results from the 2D and 3D analyses. The analytical solutions slightly underestimate the hoop force and overestimate the hoop moment compared to the FE analyses.

**Fig. 12:**
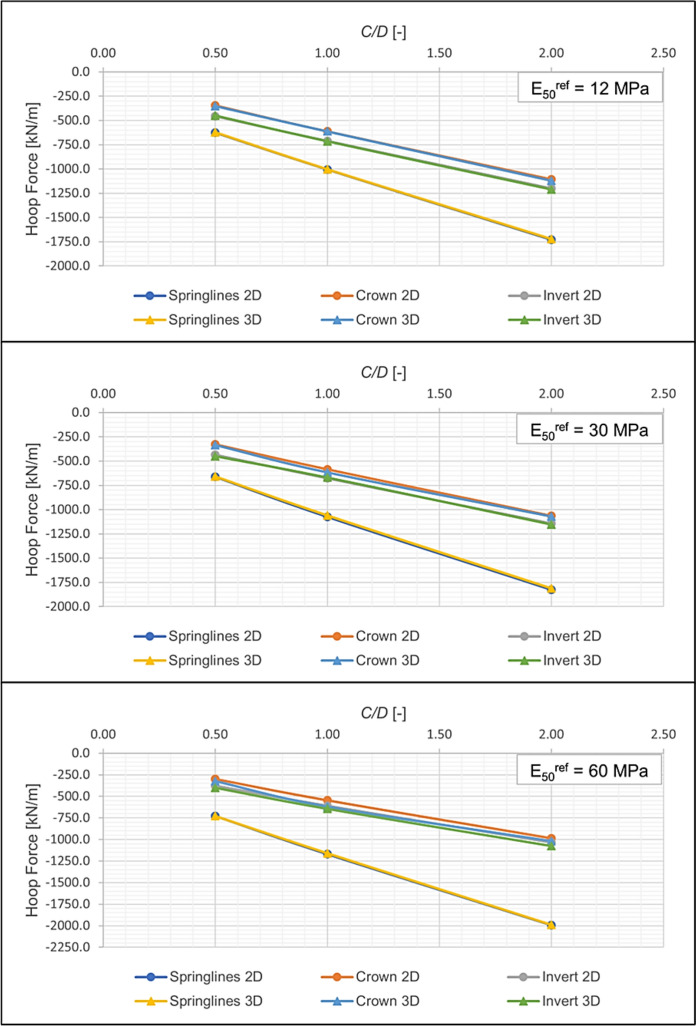
Comparison of hoop forces from 2 and 3D analyses

**Fig. 13:**
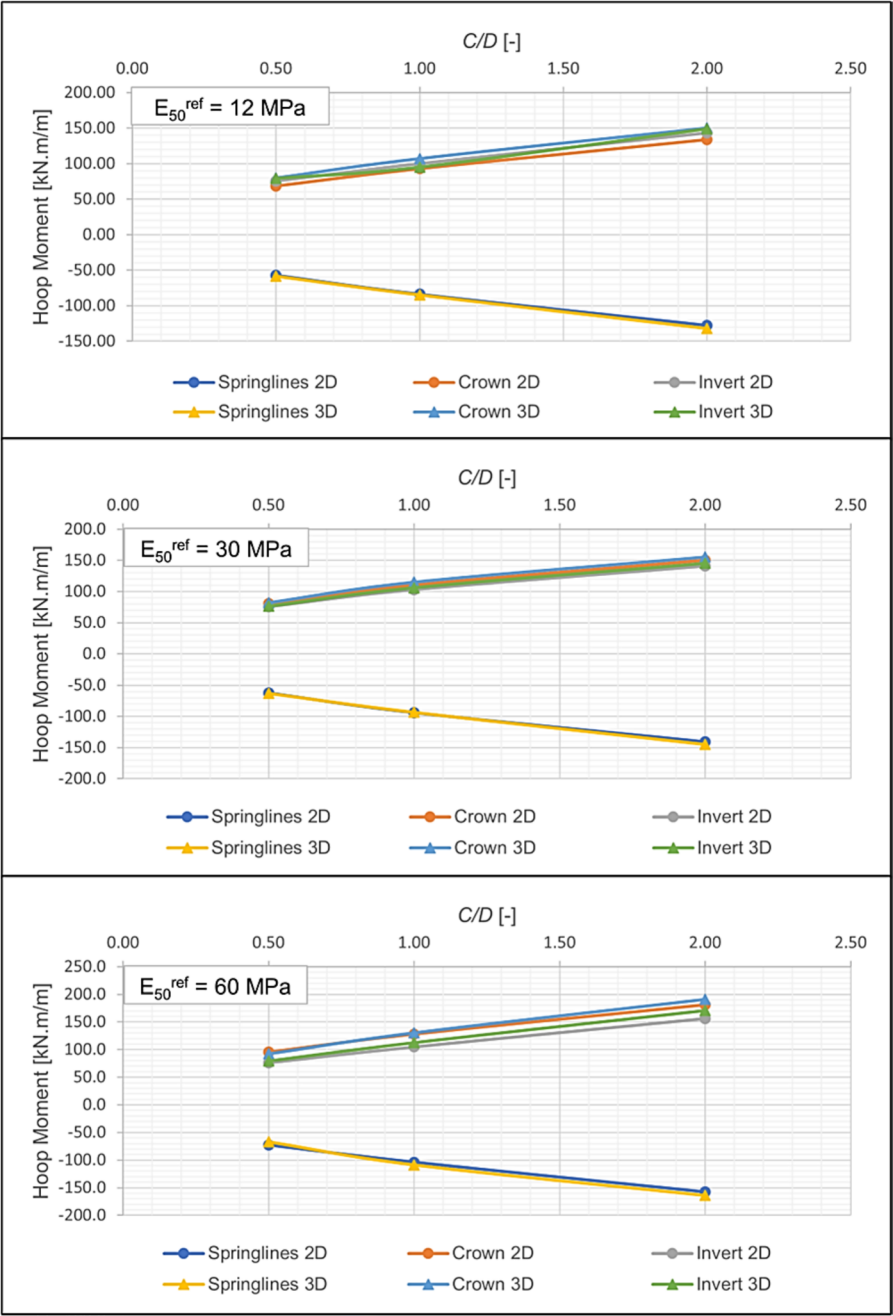
Comparison of hoop moments from 2 and 3D analyses

Furthermore, a comparison of 2D and 3D FE results for different C/D ratios and soil strengths, as shown in Figs. 12 and 13, indicate that increasing the C/D ratio leads to a linear increase in lining stresses. This implies that the ratio of forces/moments before and after CP breakout will yield similar results for different C/D ratios. It should be noted, however, that this conclusion applies only to shallow tunnels and may not hold for deep tunnels. Based on this observation, a C/D ratio of 1 was chosen for the parametric analyses.

### Effect of Parent Tunnel Volume Loss on Stress Regime Around Circular CP

The subsequent sections examine the impact of volume loss in the PT lining on the stresses around a circular CP opening. Different magnitudes of PT lining volume loss are analysed to evaluate their effect on ground–structure interaction and stress redistribution around the CP opening.

#### Effect at Springlines

**Fig. 14:**
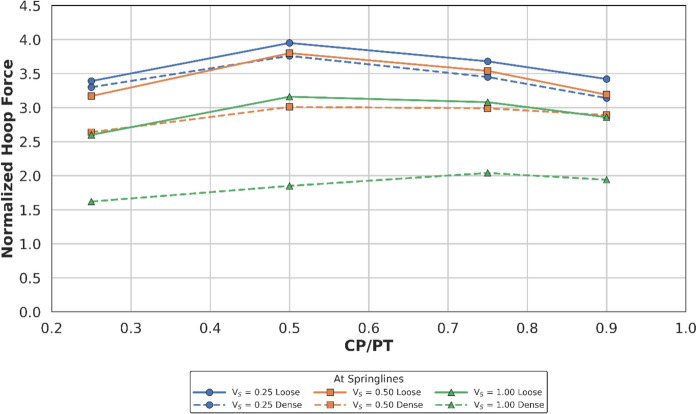
Normalised hoop force at springlines of CP with varying CP/PT ratio

**Fig. 15:**
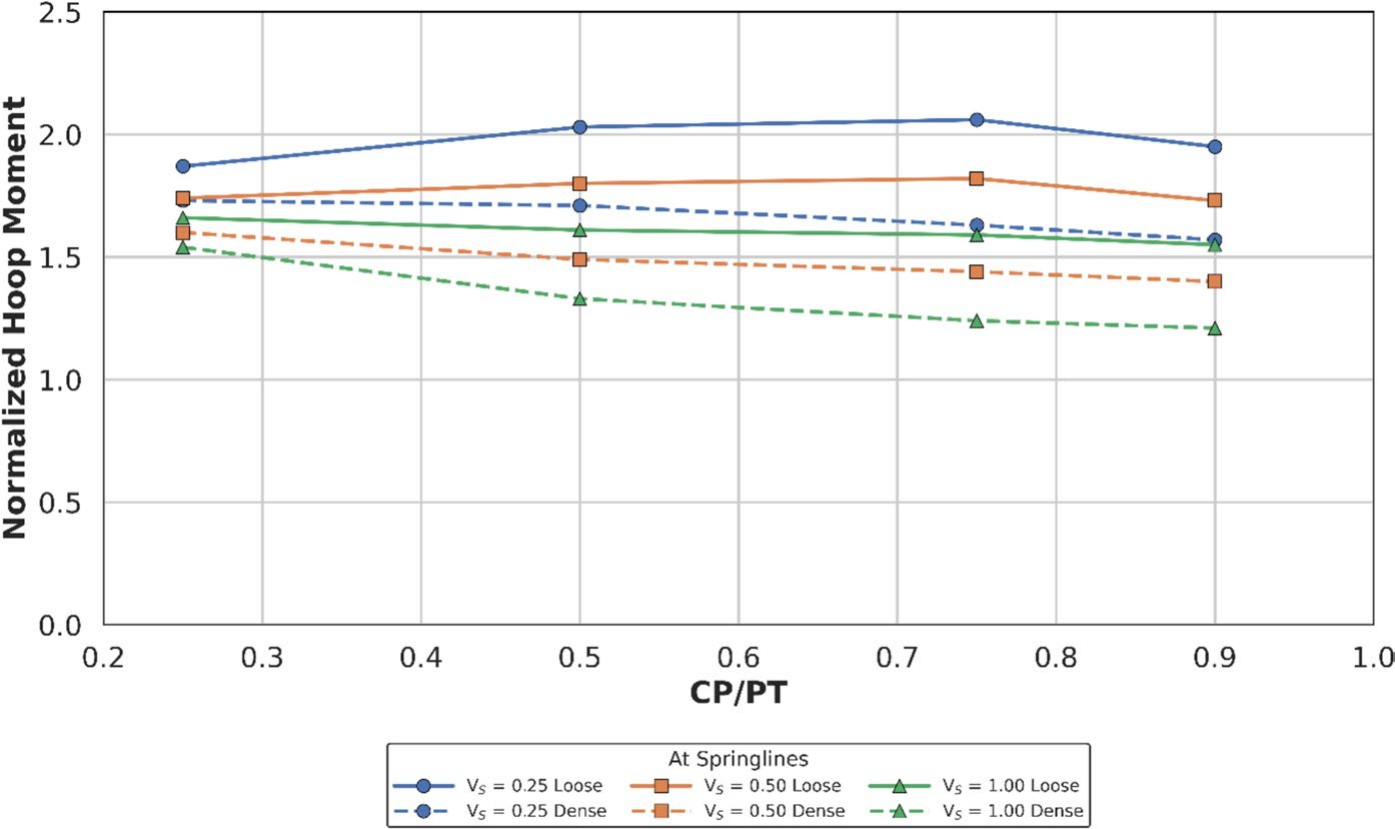
Normalised hoop moment at springlines of CP with varying CP/PT ratio

**Fig. 16:**
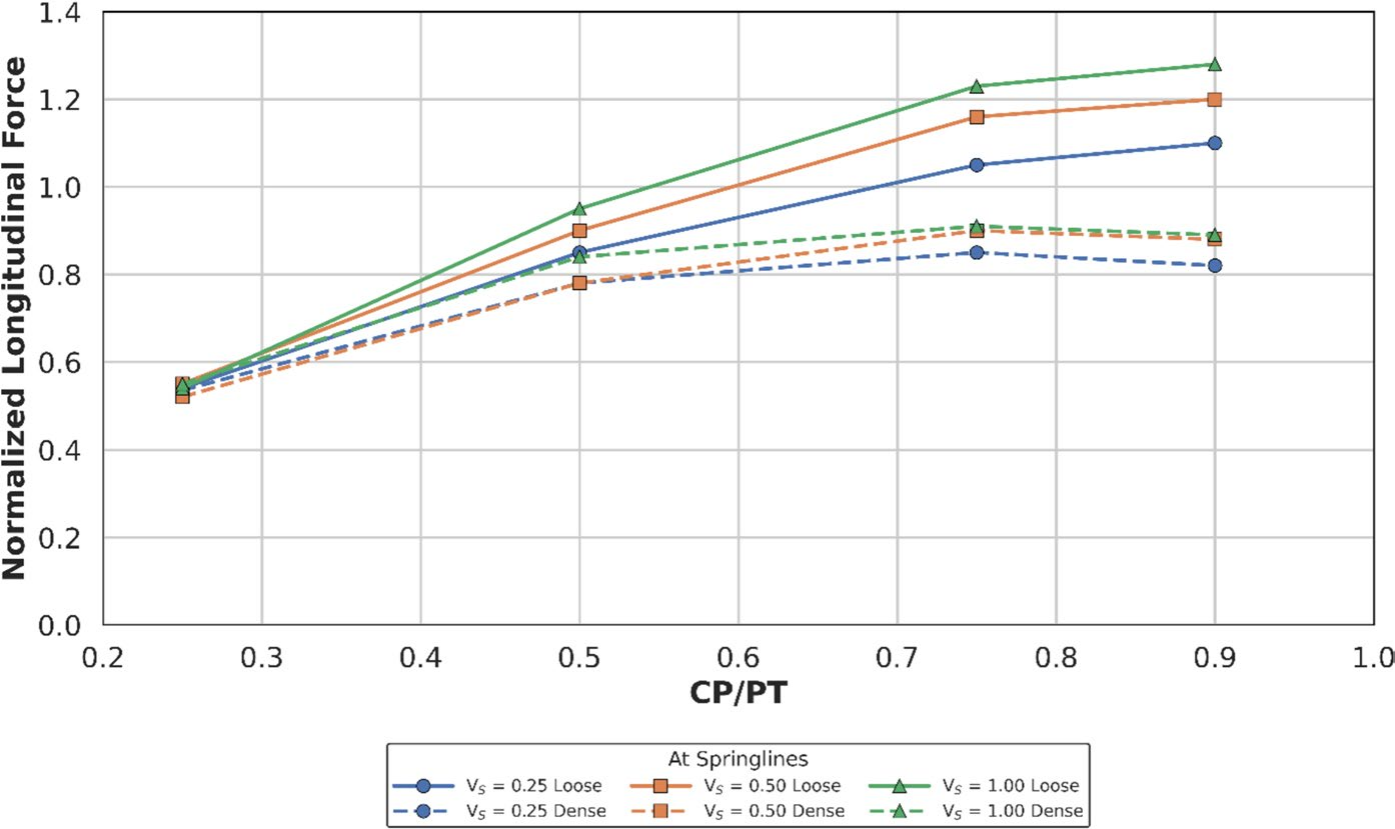
Normalised longitudinal force at springlines of CP with varying CP/PT ratio

**Fig. 17:**
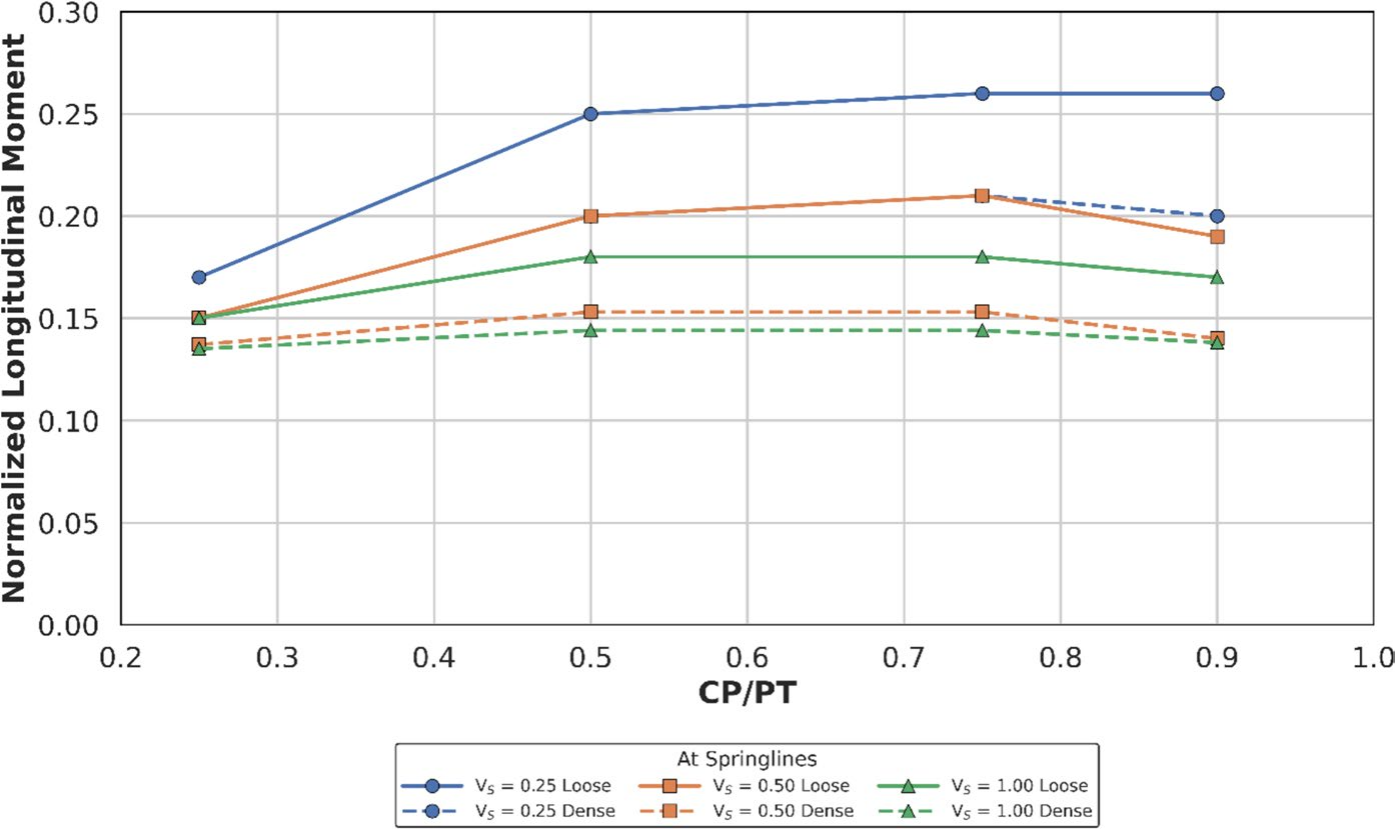
Normalised longitudinal moment at springlines of CP with varying CP/PT ratio

Figures 14 and 15 show the increase in normalised hoop force and moment around the CP springlines, while Figs. 16 and 17 illustrate the change in normalised longitudinal force and moment around the CP springlines. It is evident that for any given soil strength, with increase in PT volume loss, the residual long-term stress on the lining decreases. The reduction in lining stress is an outcome of increased radial deformation, corresponding to the applied surface contraction representing the volume loss.

This leads to hoop forces and hoop/longitudinal bending moments at the springlines of the CP decrease as the PT volume loss increases, while interestingly the longitudinal forces increase. The maximum increase in hoop force and moment occurs if the CP/PT ratio is equal to 0.5, however with increase in PT volume loss, the maximum values shift to CP/PT ratio of 0.75 and 0.25 for hoop force and bending moment respectively.

A similar trend is observed for longitudinal moments, but these are negligible magnitude wise (Fig. 17). An almost linear increase in magnitude is observed for longitudinal forces in loose soils, while for denser soils, longitudinal forces tend to plateau after a CP/PT ratio of 0.75 (Fig. 16). Maximum longitudinal force increase is observed at a CP/PT ratio of 0.95 for all densities of soil and a steady increase is observed with an increase in PT volume loss. It is also clear that the springlines of the CP remain under compression as all the normalised values are positive.

##### Effect at Crown

**Fig. 18:**
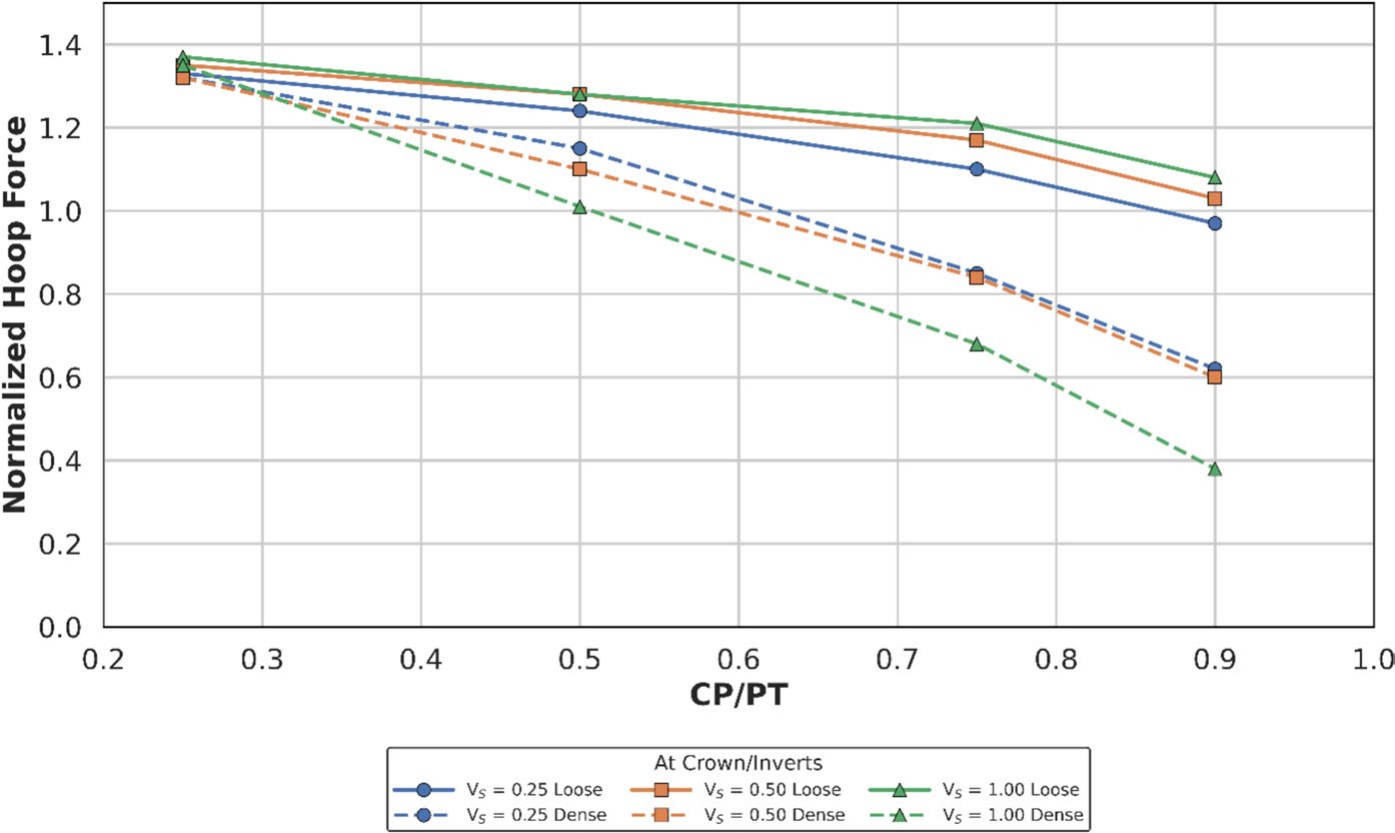
Normalised hoop force at crown/invert of CP with varying CP/PT ratio

**Fig. 19:**
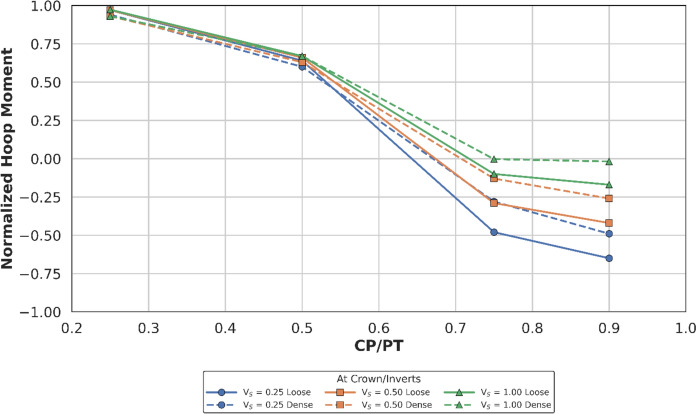
Normalised hoop moment at crown/invert of CP with varying CP/PT ratio

Figures 18 and 19 illustrate the distribution of normalised hoop forces and moments at the crown/invert of the CP. These show a linear decrease in magnitude with increasing CP/PT ratio, while hoop moments show a shift from compression to tension. An increase in PT volume loss causes a larger difference between hoop forces in loose and dense soils as evident in Fig. 18. While hoop moments show higher variance with increasing volume loss only at larger CP/PT ratios (CP/PT > 0.5).

**Fig. 20:**
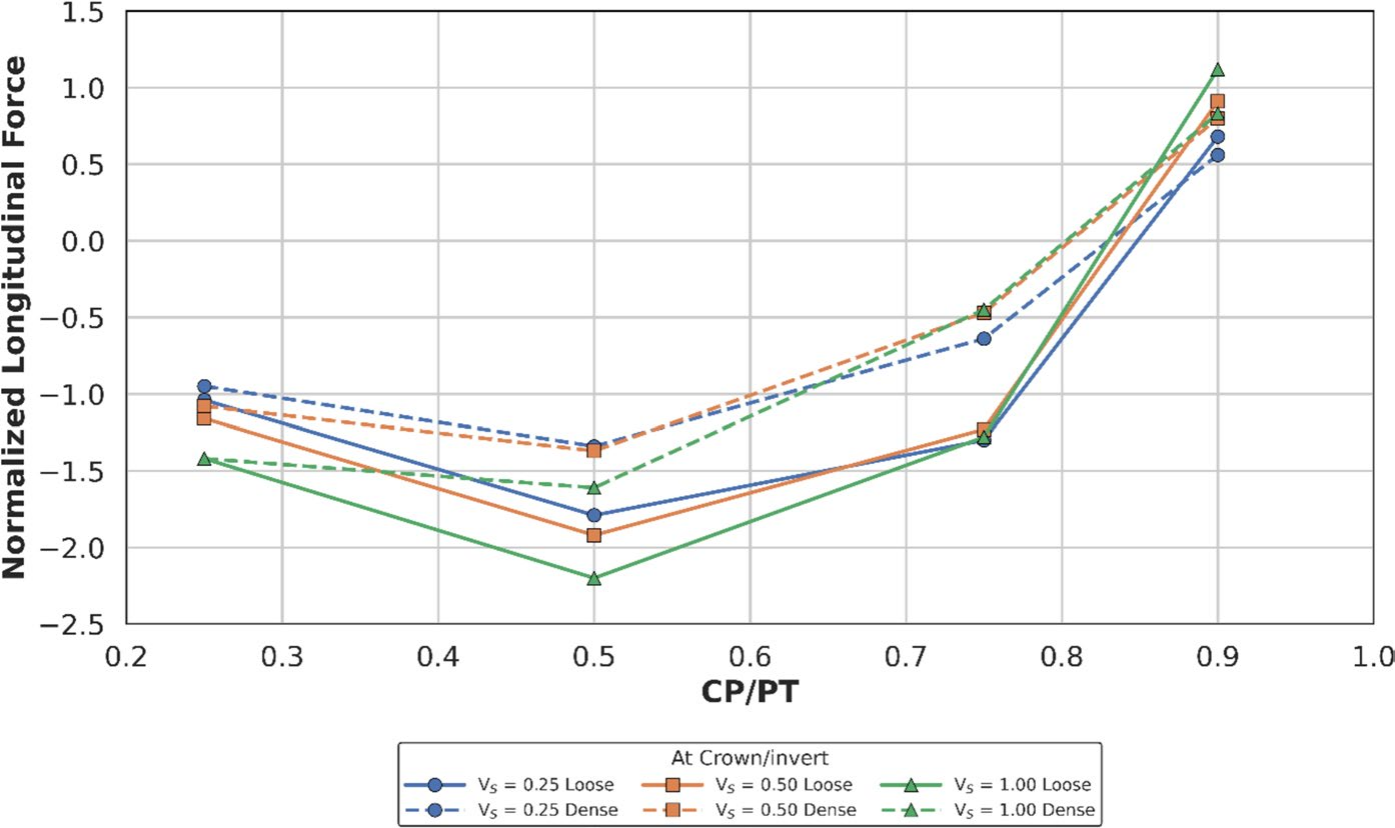
Normalised longitudinal force at crown/invert of CP with varying CP/PT ratio

**Fig. 21:**
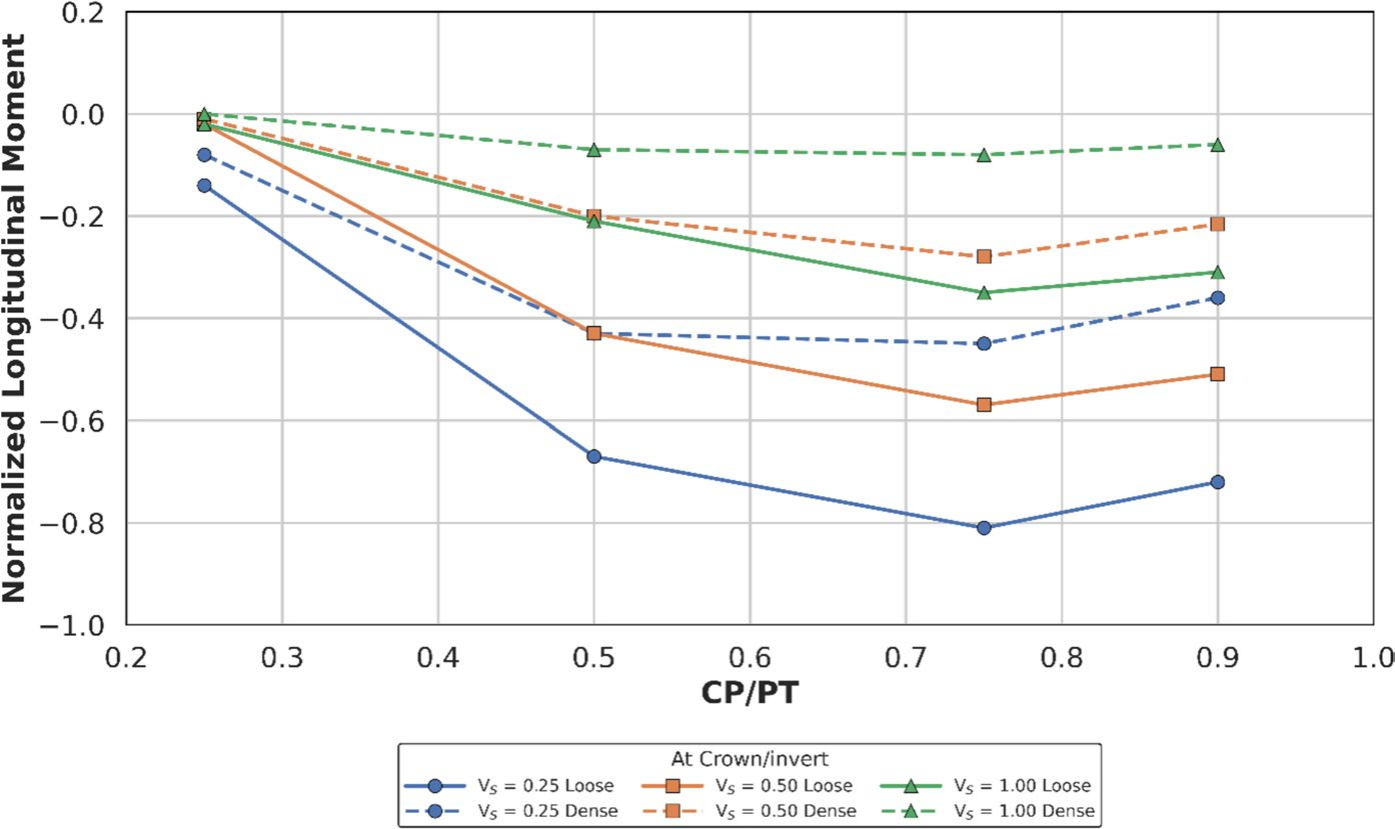
Normalised longitudinal moment at crown of CP with varying CP/PT ratio

Longitudinal forces at the crown/invert of the CP are mostly tensile, only excluding CP/PT = 0.9 which show compressive values as shown in Fig. 20. Longitudinal moments are tensile and increase with an increase in CP/PT and stabilize after CP/PT = 0.75. PT volume loss has negligible effect on longitudinal forces, but moments increasingly become tensile as volume loss decreases (refer to Fig. 21).

Similar to the stress regime at the springlines of the CP, a general decrease in magnitude for hoop and longitudinal stresses is observed with increasing soil strength. Negative normalised values indicate that the crown/invert area is predominantly in tension both in hoop and longitudinal directions, except for hoop forces, which are compressive.

#### Effect of CP Geometry on the Stress Regime Around CP

The following sections examine the impact of CP shape and volume loss, due to PT lining excavation, on the stresses around a CP opening. The CP shapes considered are illustrated in Fig. 4 and different magnitudes of PT lining volume loss are applied to evaluate its effect on the stresses around the CP opening.

#### Effect of CP Shape on the Stress Redistribution Around the Opening

**Fig. 22:**
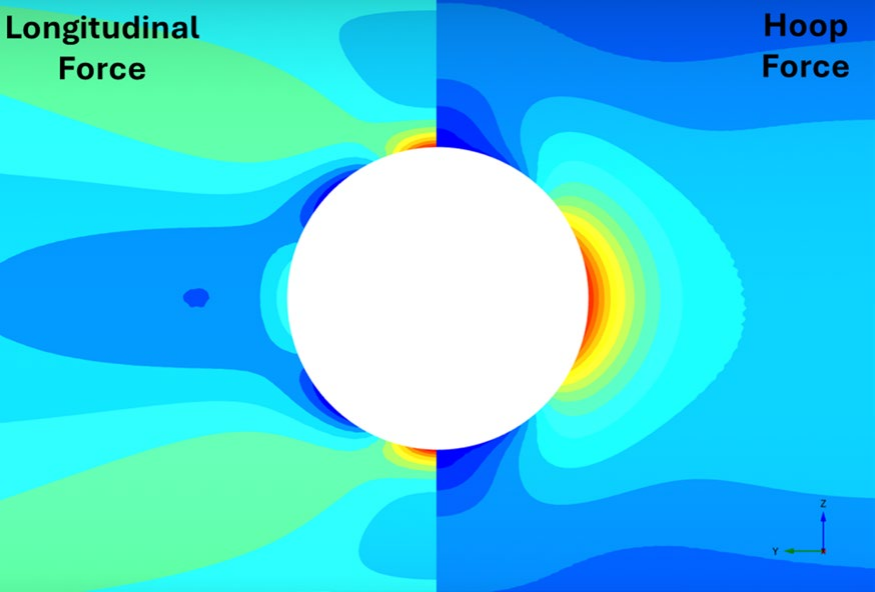
Distribution of PT lining forces around circular openings (*V*_S_ = 0.25%, CP/PT = 0.5, RD = 50%)

**Fig. 23:**
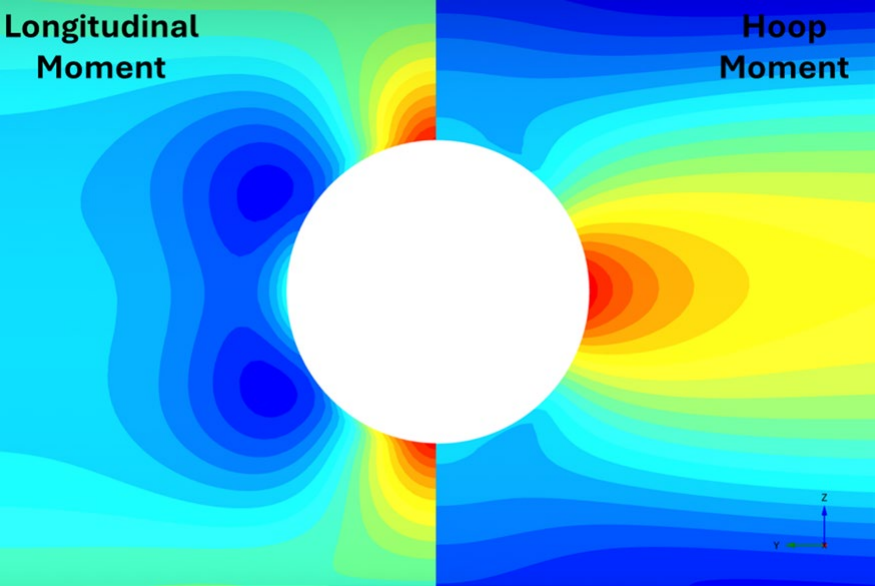
Distribution of PT lining moments around circular openings (*V*_S_ = 0.25%, CP/PT = 0.5, RD = 50%)

**Fig. 24:**
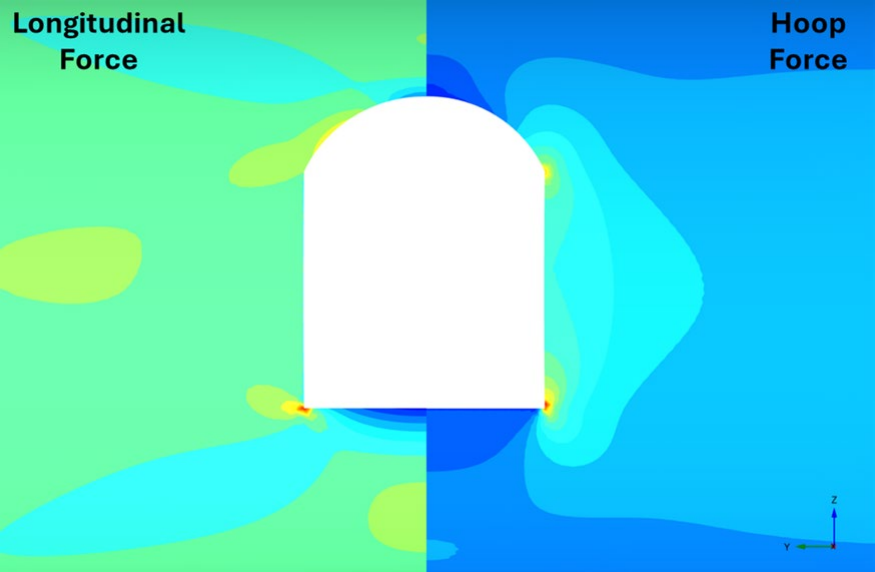
Distribution of PT lining forces around modified horseshoe openings (*V*_S_ = 0.25%, CP/PT = 0.5, RD = 50%)

**Fig. 25:**
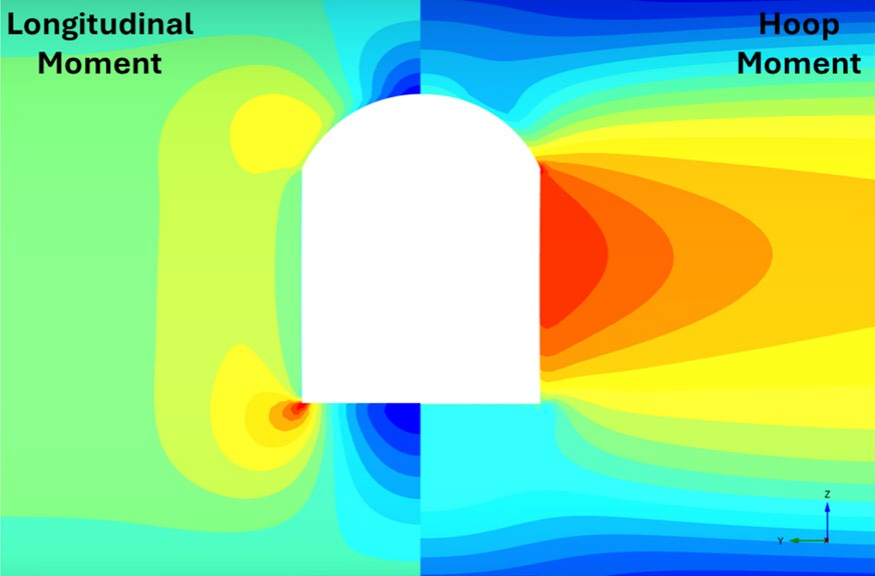
Distribution of PT lining moments around modified horseshoe openings (*V*_S_ = 0.25%, CP/PT = 0.5, RD = 50%)

**Fig. 26:**
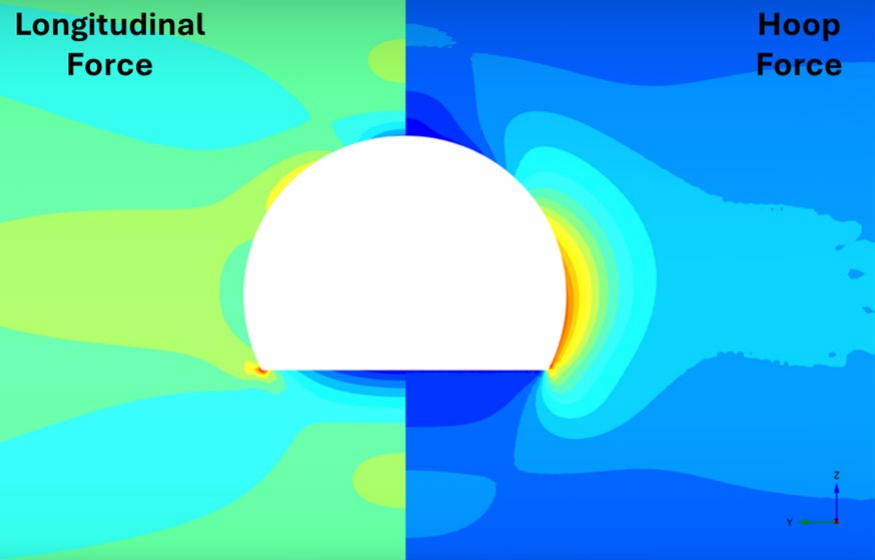
Distribution of PT lining forces around inverted-D openings (*V*_S_ = 0.25%, CP/PT = 0.5, RD = 50%)

**Fig. 27:**
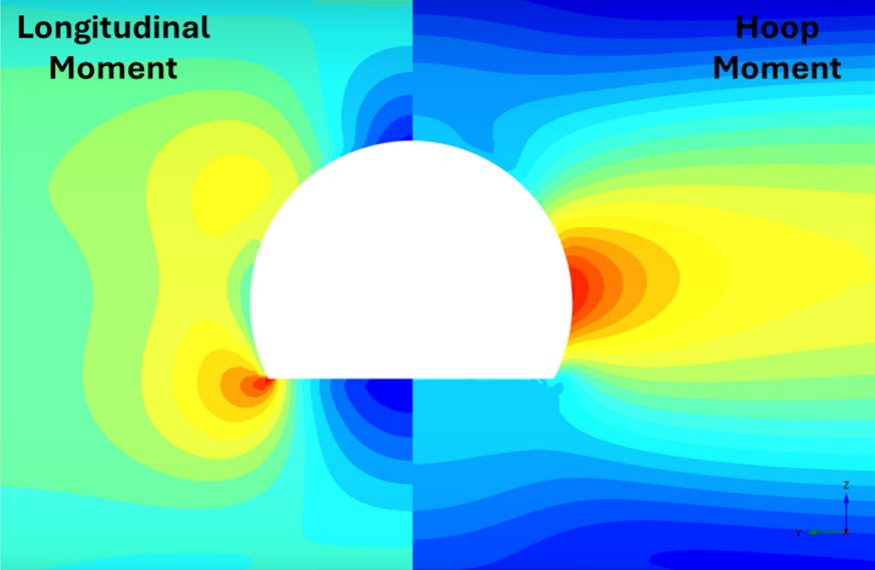
Distribution of PT lining moments around inverted-D openings (*V*_S_ = 0.25%, CP/PT = 0.5, RD = 50%)

**Fig. 28:**
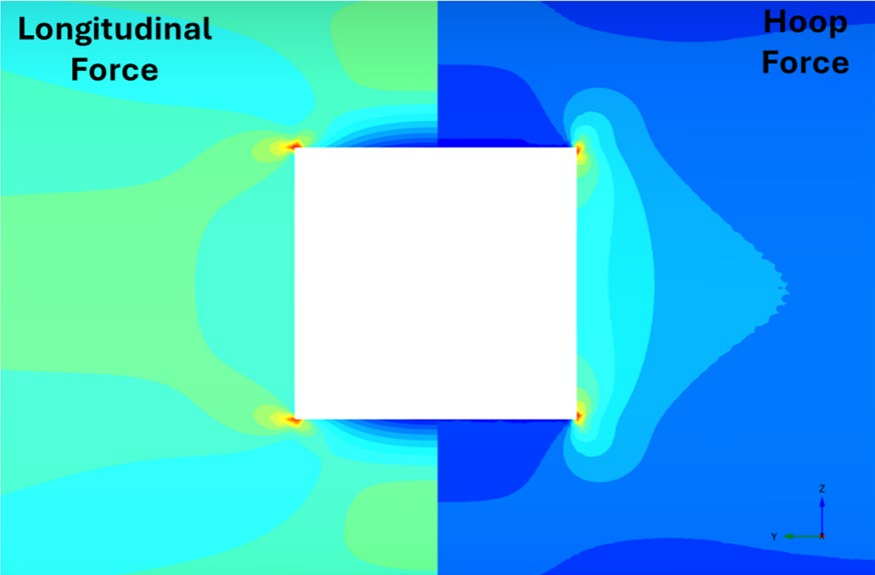
Distribution of PT lining forces around square openings (*V*_S_ = 0.25%, CP/PT = 0.5, RD = 50%)

**Fig. 29:**
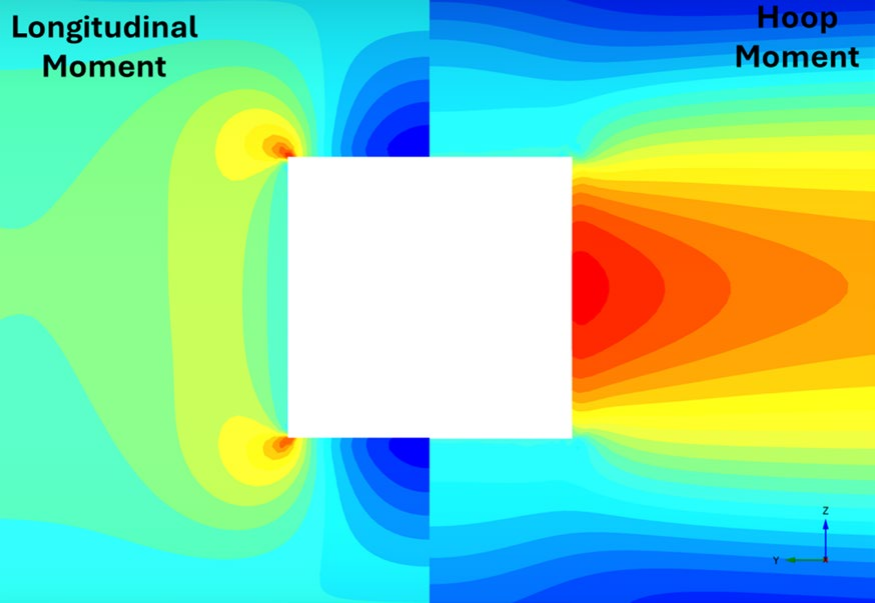
Distribution of PT lining moments around square openings (*V*_S_ = 0.25%, CP/PT = 0.5, RD = 50%)

Figures 22, 23, 24, 25, 26, 27, 28 and 29, illustrate the stress distribution PT lining internal forces and moments around the CP opening. For a circular CP, it is evident that compressive hoop stresses are concentrated at the springlines of the opening while tensile longitudinal stresses show concentrations at the crown and invert, with magnitude reducing drastically near the springlines of the opening. Circular shapes are in general excellent in transferring stresses around the opening by virtue of stress arching.

For modified horseshoe and inverted-D shapes, the maximum compressive hoop stresses occur at the springlines of the opening with very high stress concentrations at the corners near the crown and invert as well. Tensile longitudinal stresses concentrate near the invert and crown while some compressive stresses near the shoulder and invert of the opening. For square openings, maximum stresses (both hoop and longitudinal) are found near the corners of the opening, exhibiting least stress arching among the shapes analysed.

#### Effect of CP Shape on PT Lining Hoop Stresses

**Fig. 30:**
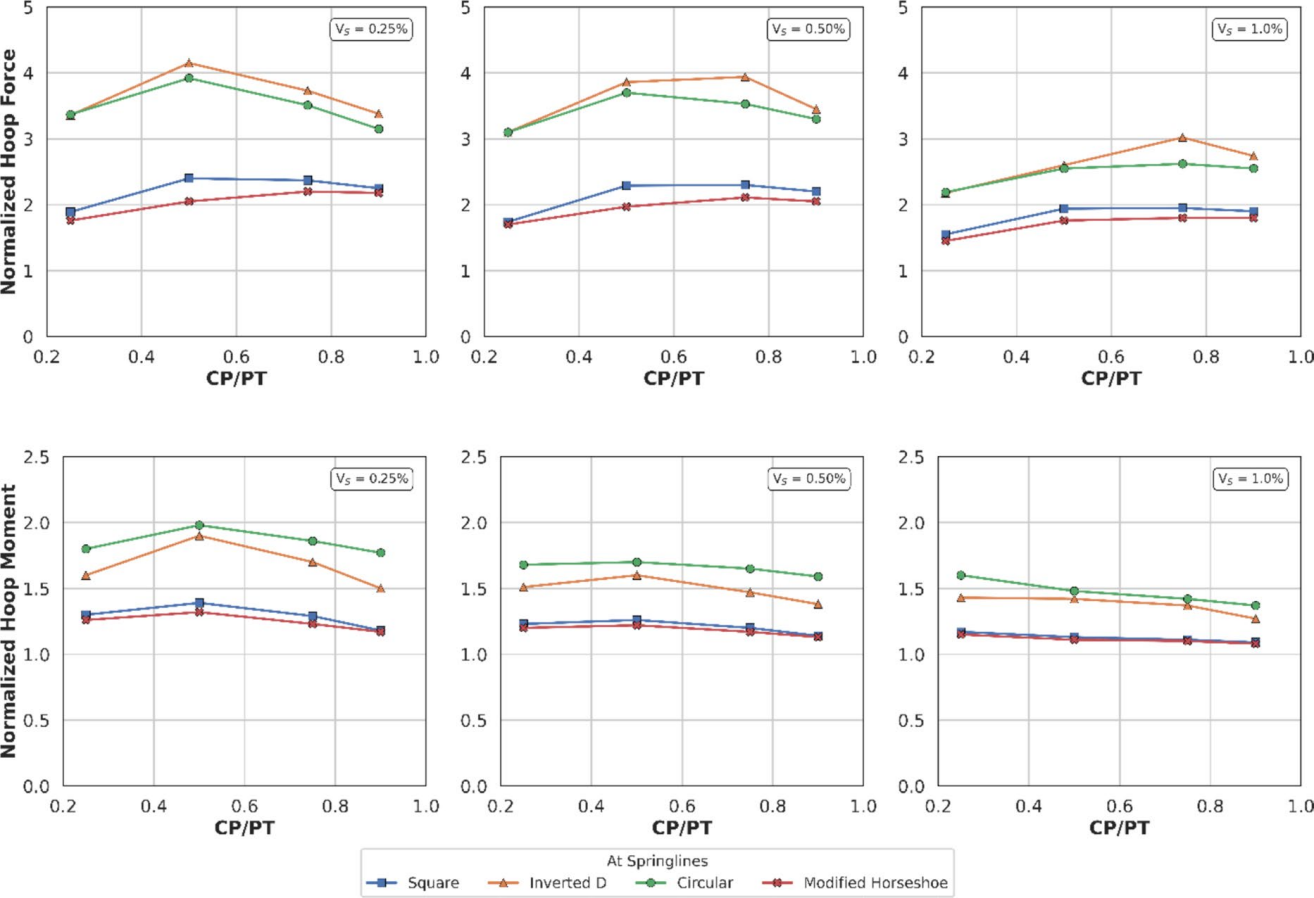
Normalised hoop stresses at the springlines of CPs with different shapes

**Fig. 31:**
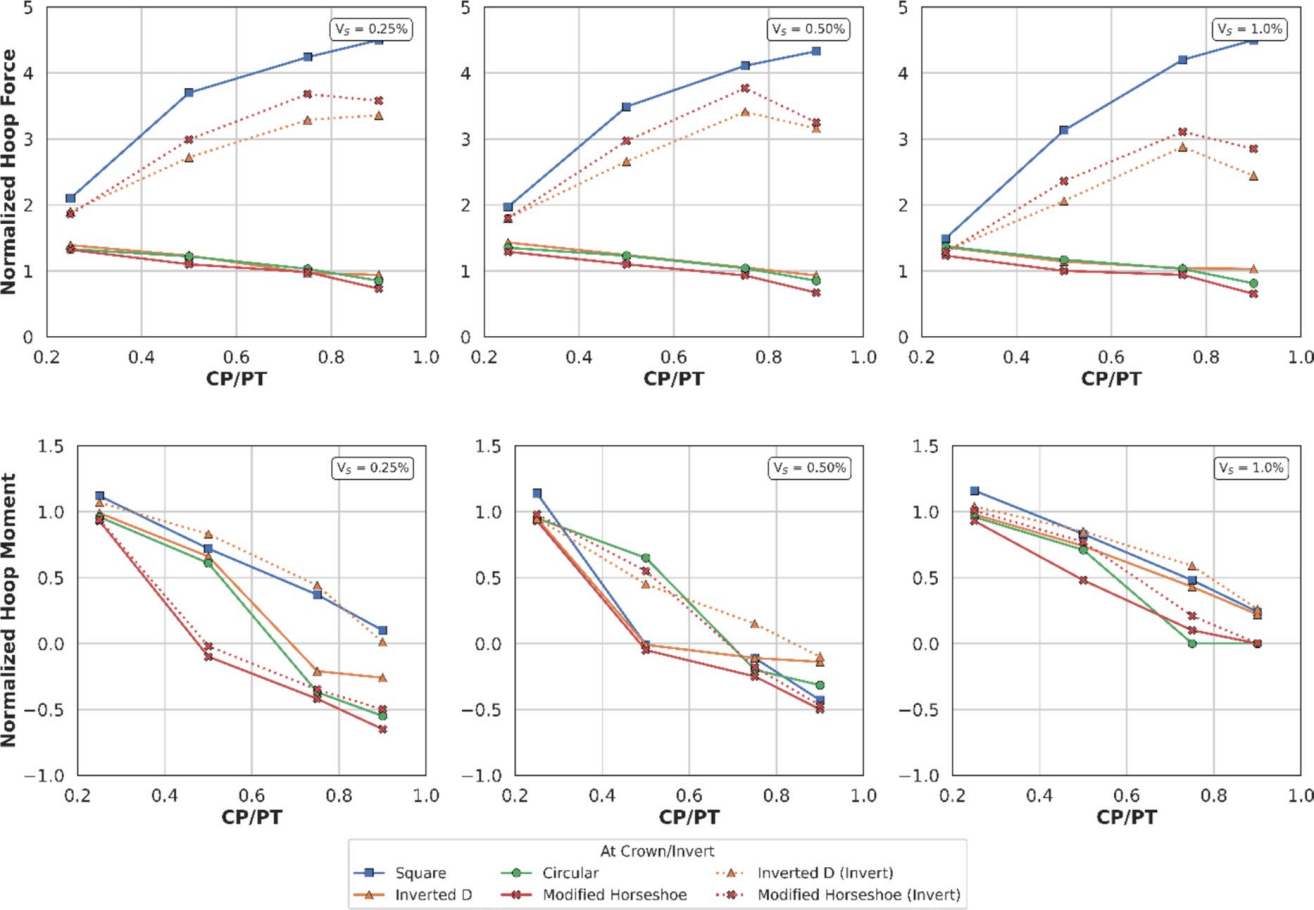
Normalised hoop stresses at the crown/invert of CPs with different shapes

From Figs. 30 and 31, it can be observed that at the springlines of the CP, circular and inverted-D shaped CPs produce the highest hoop stresses while square and modified horseshoe shaped CPs produce a lower concentration of hoop stresses. This can be attributed to stress arching occurring in circular and inverted-D shapes which efficiently transfer overburden stresses from the crown to the springlines of the CP. High stresses are observed at the crown/invert of the square CP and invert of the modified horseshoe and inverted-D shapes.

Hoop moments at the crown/invert of the CPs can be neglected as their magnitude is insignificant while the main design driver for the different shapes of CP are hoop stresses at the springlines and hoop forces at the invert of CP shapes other than circular.

#### Effect of CP Shape on PT Lining Longitudinal Stresses

**Fig. 32:**
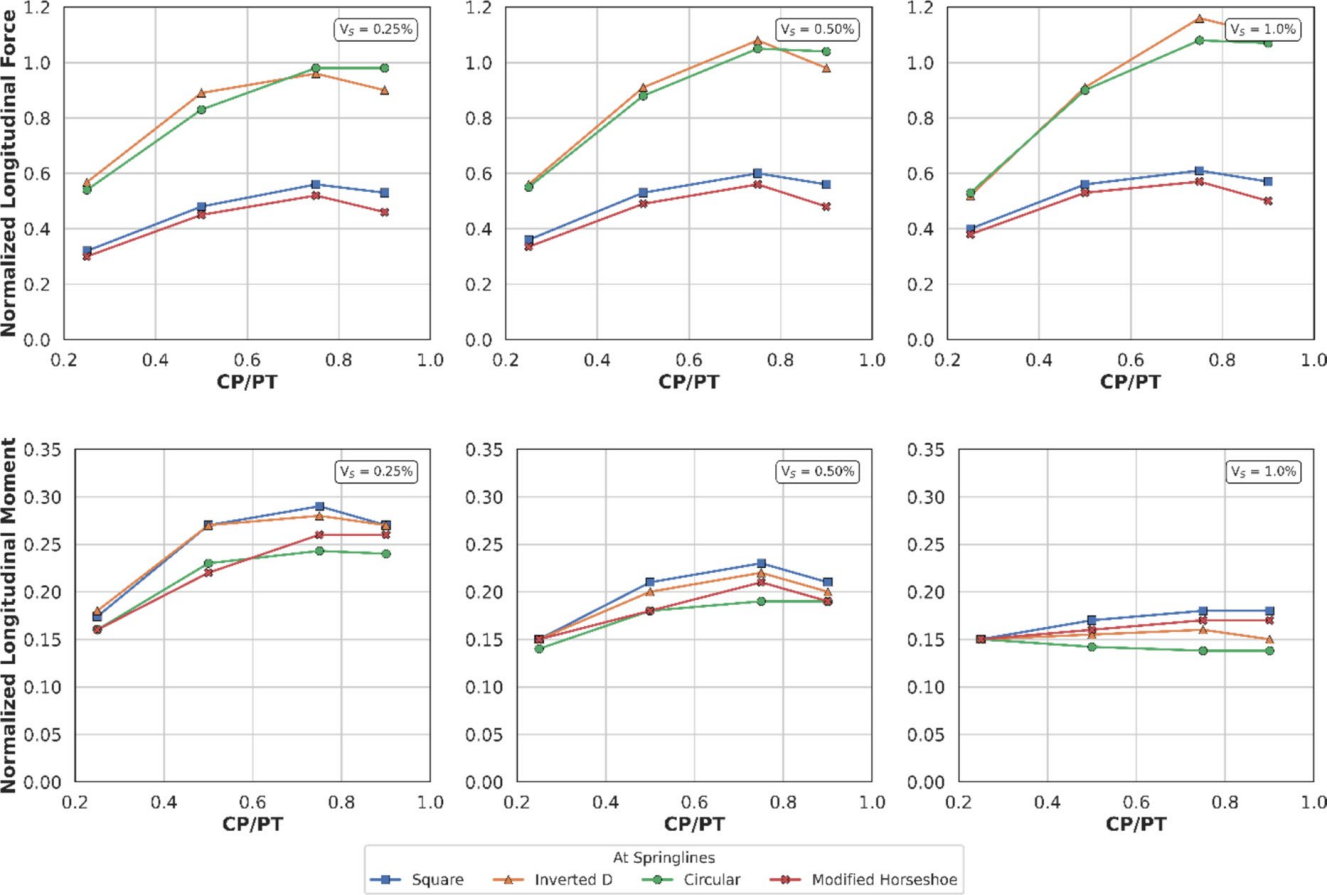
Normalised longitudinal stresses at the springlines of CPs with different shapes

**Fig. 33:**
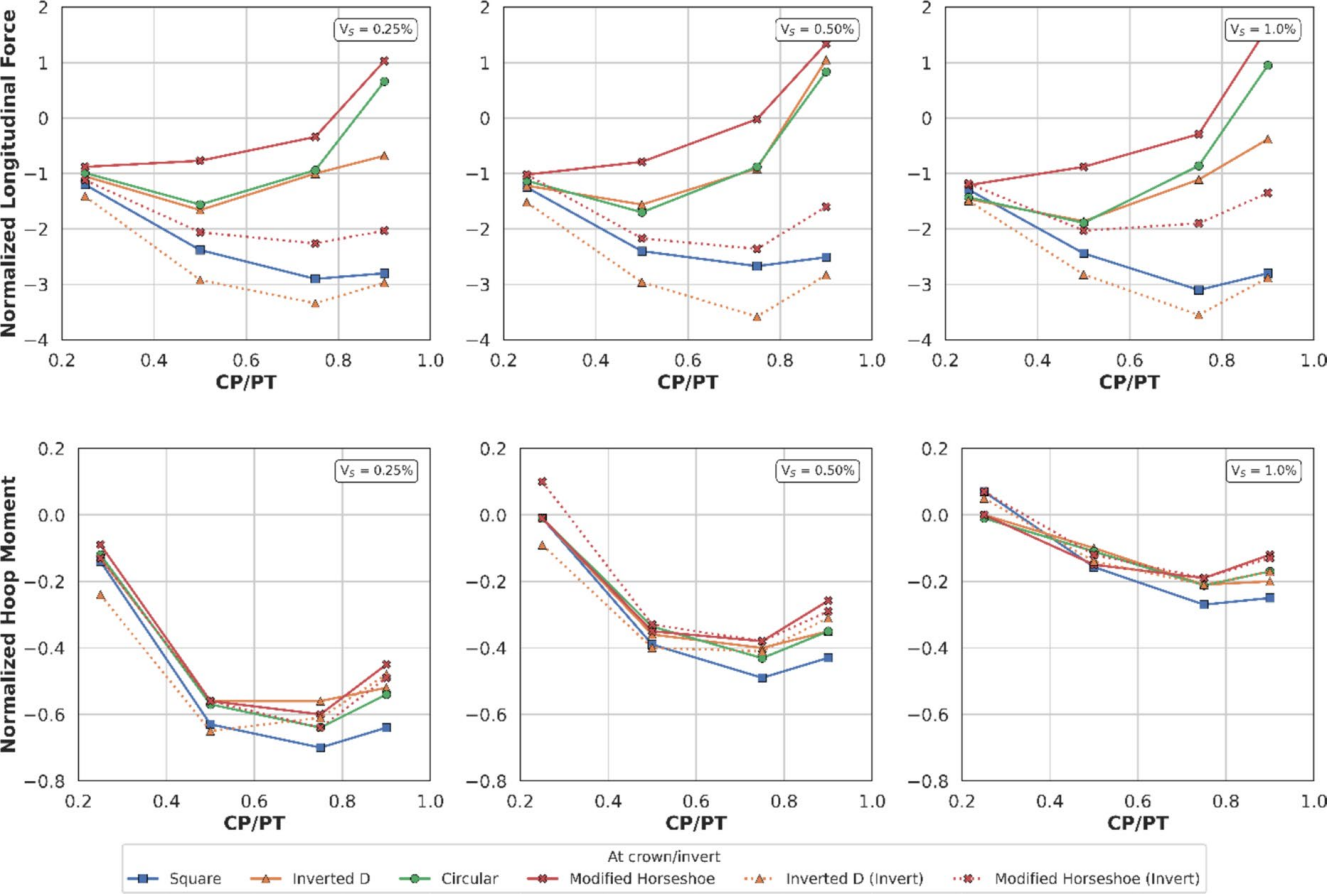
Normalised longitudinal stresses at the crown/invert of CPs with different shapes

Figures 32 and 33 show longitudinal stresses at the springlines and crown/invert of the different CP geometries. Normalised longitudinal stresses can be observed to have negligible values (mostly less than one) at the springlines of the CPs. Crown/invert of square CP and the inverts of modified horseshoe and inverted-D CPs show highest tensile longitudinal stresses. Least values are observed in circular CP while inverts of modified horseshoe and inverted-D CPs generate very high longitudinal stresses.

### Effect of CP Geometry on its Deformation Response

**Fig. 34:**
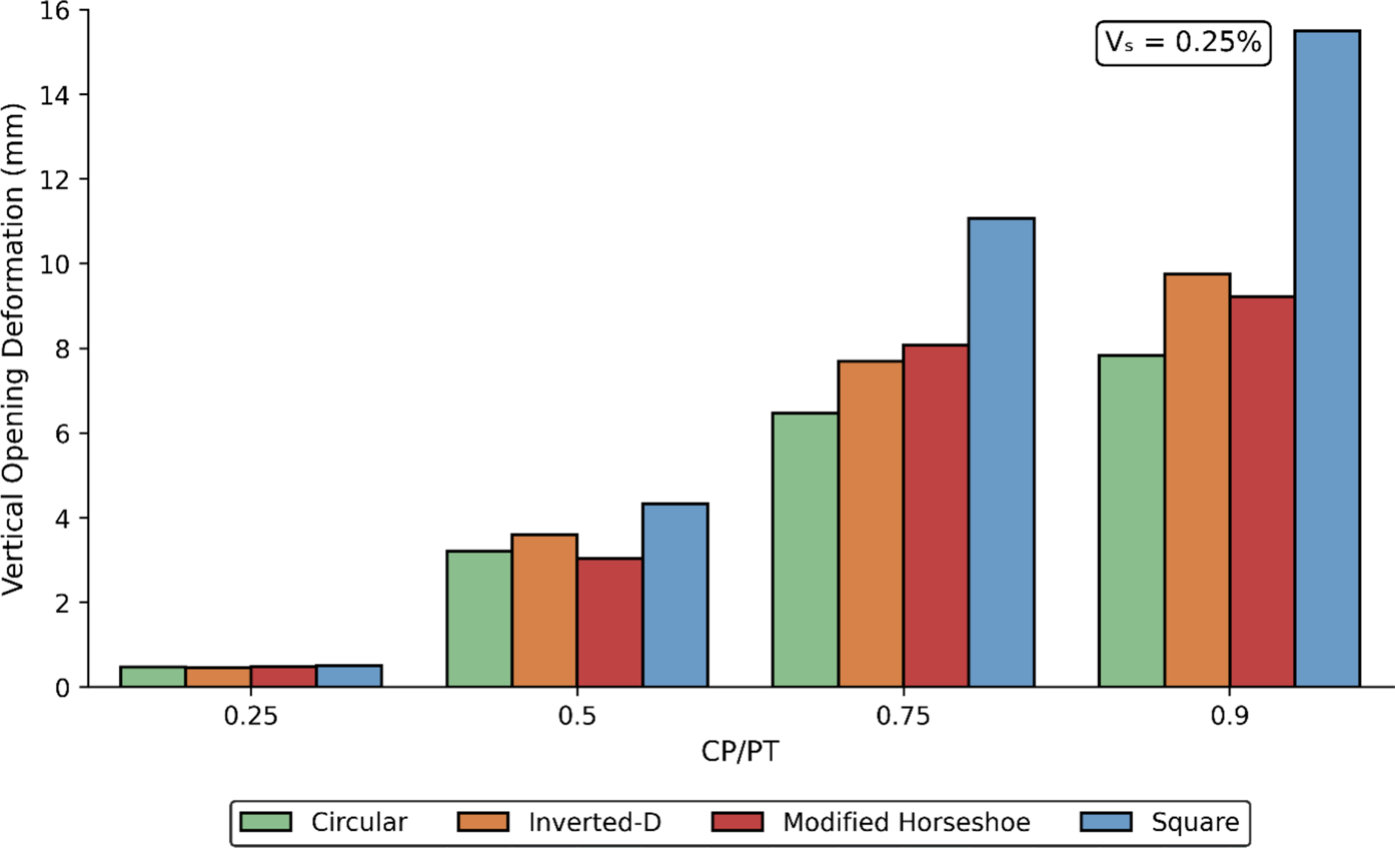
Vertical CP Deformation at PT volume loss of 0.25% with different opening shapes and sizes

**Fig. 35:**
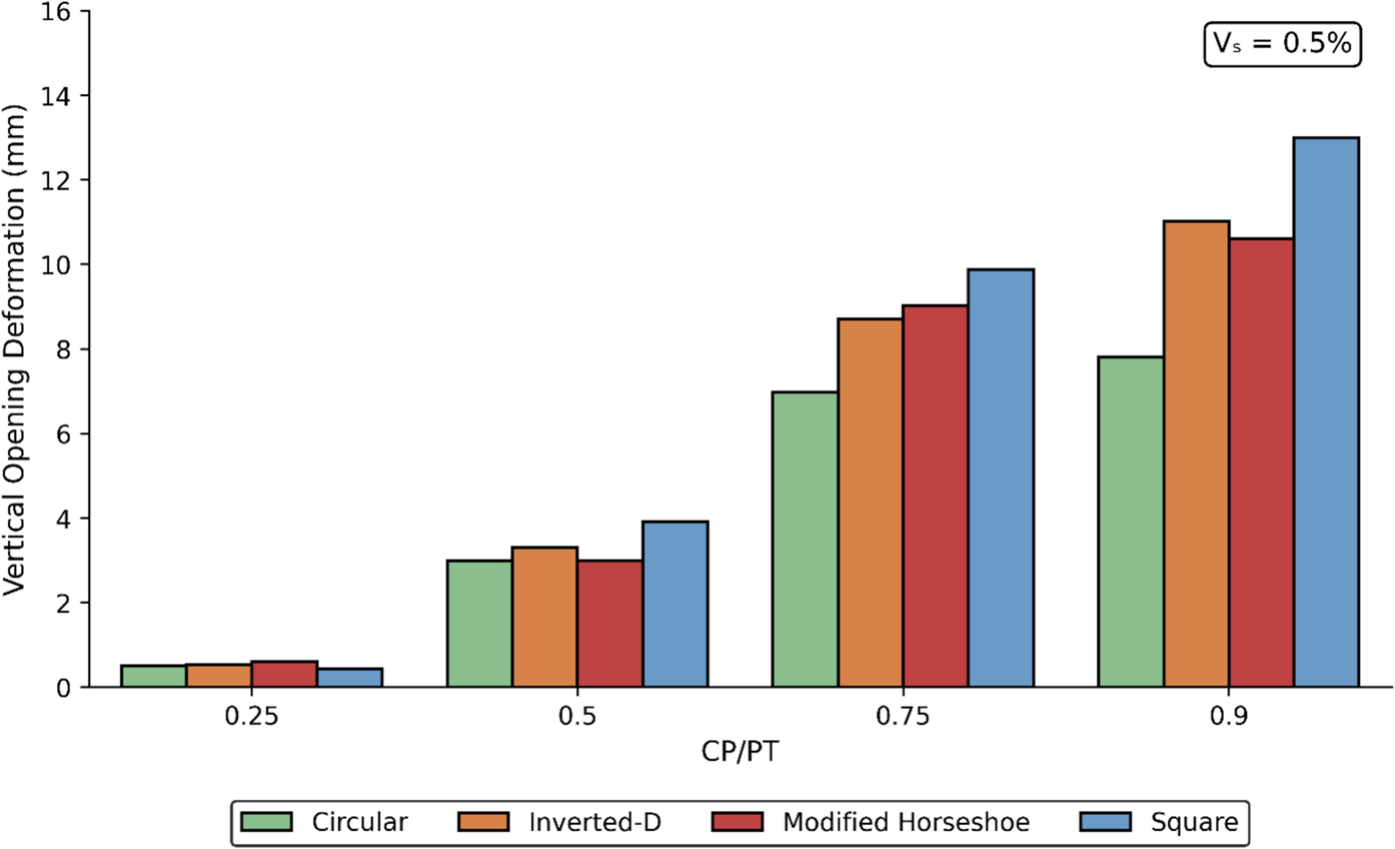
Vertical CP Deformation at PT volume loss of 0.5% with different opening shapes and sizes

**Fig. 36:**
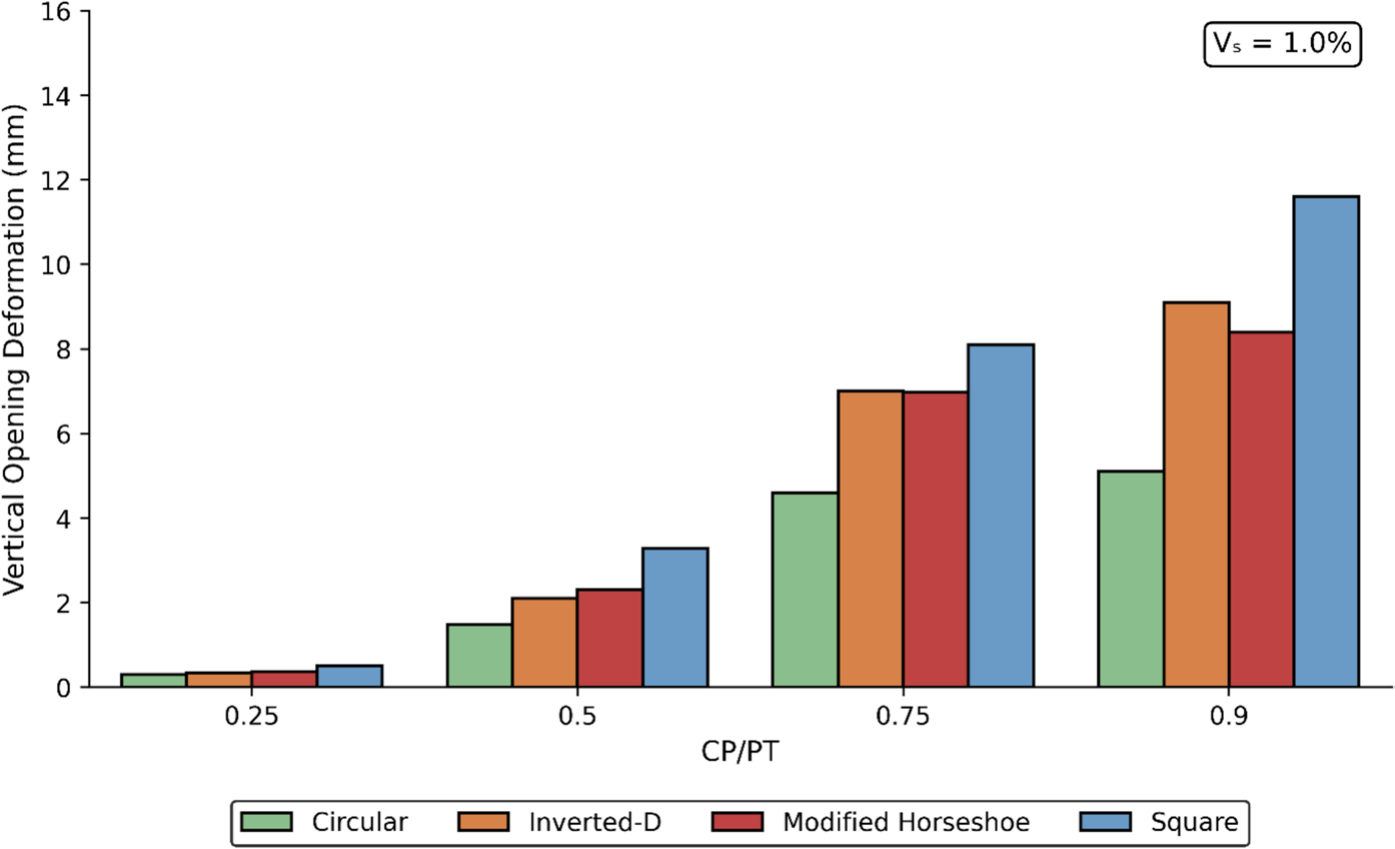
Vertical CP Deformation at PT volume loss of 1.0% with different opening shapes and sizes

The opening deformations result from stress redistribution in the surrounding ground following excavation. These movements are influenced by several factors, including the mechanical properties of the soil or rock, in-situ stress conditions, excavation geometry, and the type and sequence of applied support measures. Vertical CP deformations were recorded for different CP shapes, sizes, and prescribed PT volume loss values. The results are illustrated in Figs. 34, 35 and 36.

The results indicate that vertical deformations decrease with increasing PT volume loss, as the residual stresses acting on the CP opening are progressively reduced. Among the geometries analysed, the square CP exhibited the largest vertical deformations, whereas the circular CP consistently demonstrated the smallest.

Overall, the circular CP provides the greatest structural stability, experiencing the lowest vertical deformations when compared with the other geometries. This behaviour can be attributed to stress arching around the circular opening, which reduces stress concentrations at the boundary and limits the magnitude of stresses acting directly on the opening.

## Discussion of Results

**Fig. 37:**
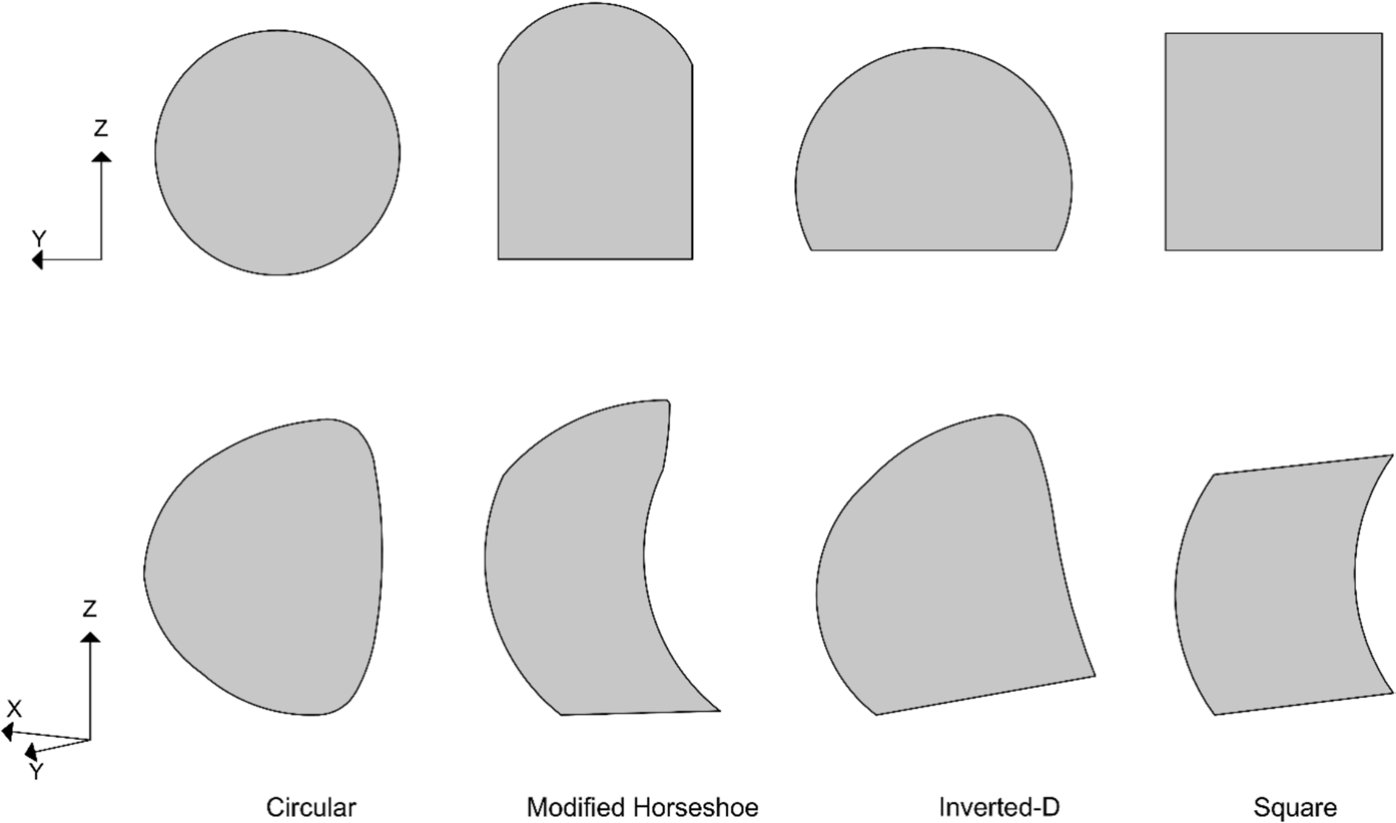
CP opening distortion when projected on the PT curved surface (CP/PT = 0.90)

From the results presented in this study, it is evident that the shape of the cross passage plays a critical role in governing stress transfer within the parent tunnel lining. Spyridis and Bergmeister (2015) briefly discussed the geometrical effects associated with lateral tunnel openings, emphasizing that the shape of the opening corresponds to the projection of the CP cross-section onto the curved surface of the parent tunnel. This phenomenon is illustrated in Fig. 37, where the distortion becomes more pronounced as the CP/PT ratio increases, making stress arching more complex and less straightforward to define. In particular, for the modified horseshoe opening, a distinct notch develops at the crown, creating an additional point of stress concentration that may influence the local structural response. Similar phenomenon is observed for the circular and inverted-D shapes as well but to a lesser extent.

The normalised force–moment plots developed in this study provide practical design aids for tunnel designers at feasibility stage. These plots can be applied once the hoop force and hoop moment at the springlines of the parent tunnel have been determined, which can be readily obtained from a 2D plane-strain analysis. The hoop force at the springlines of the parent tunnel can be used to estimate the corresponding forces around the cross passage opening, while the hoop moment can be employed to determine the associated bending moments in the vicinity of the opening.

Hoop stresses at the springlines and longitudinal stresses at the crown/invert of the CP are of utmost importance for the design of lining near CPs, since the magnitude is significant, compared to the hoop stresses at ring closure (before the introduction of CP). Longitudinal stresses at the springlines and hoop stresses at the crown/invert of the CP are negligible, hence these can be ignored for shallow tunnels.

Initial volume loss due to PT excavation also plays a major role in determining the residual applied stress on the PT lining, which determines the magnitude and location of stress concentrations around the CP opening. At springlines of the CP, an increase in PT volume loss increases longitudinal force only while hoop stresses and longitudinal moment decrease. At crown/invert of CP, only hoop force increases with increase in PT volume loss, all other forces and moments decrease.

For instance, the normalised hoop force at the springlines of a circular CP reduces from a maximum of 3.95 to 3.2 (CP/PT = 0.5), which is a 19% reduction. Similarly, normalised longitudinal force increases from a minimum of 0.82 to 1.8 (CP/PT = 0.90) resulting in an increase of 15%.

Most of the peak values occur at approximately CP/PT = 0.5, beyond which the forces tend to plateau (longitudinal forces) or reduce (hoop forces). This behaviour is attributed to the increasing deformability of the opening with size. As the opening becomes larger, it undergoes greater deformation, and consequently, results in lower forces to be transferred to the lining.

Furthermore, CP geometry significantly influences the redistribution of stresses within the PT lining around the opening, both in terms of magnitude and the spatial distribution of stresses. For example, although the hoop forces and moments at the springlines of a circular CP are greater than those of a square CP, the square CP exhibits nearly a fivefold increase in hoop stress at its crown and invert.

Similar trends are observed for the modified horseshoe and inverted-D geometries, which tend to attract pronounced stress concentrations at their invert corners. A comparable pattern is evident in the longitudinal stresses developed around the various CP configurations, indicating that CP geometry plays a critical role in governing both hoop and longitudinal stress distributions.

This shows that CP shape and PT volume loss change both where and how large lining demands become, the designer should include CP geometry as a primary design variable when specifying lining thickness and reinforcement, evaluate a realistic range of volume loss scenarios in structural checks and detail crown/invert reinforcement for square or sharp-cornered openings where stress concentrations may develop.

The normalised force-moment plots developed in this study can provide excellent design aid to practitioners at feasibility and preliminary design stages. For detailed design stage, a detailed 3D soil-structure-interaction analysis is recommended.

### Limitation and Future Extension of the Study

The present study is subject to the following limitations:

- CP lining installation was not modelled since the analysis focuses on initial stress redistribution from the opening only. Real construction sequences (temporary supports, staged excavation, TBM versus SCL, face support) may alter the magnitude and timing of stresses.
- While HSS captures nonlinearity and small-strain stiffness of the soil, the constitutive model parameters were estimated and not validated against laboratory tests. Future studies can incorporate lab testing within the scope of a numerical study.
- In the absence of site-specific monitoring data or experimental measurements, the three-dimensional numerical model was validated against established analytical solutions. Future research should aim to complement these findings through comparison with field monitoring or controlled laboratory experiments.
- The conditions considered in the current study may differ from real conditions such as layered strata, ground water presence and deep tunnels. Future studies should extend the current findings by incoporating these aspects
- Lining is assumed to be a uniform linear elastic plate element with a single modulus of elasticity. Incorporating segment joints (for segmental lining) or varying lining modulus based on application time (Sprayed Concrete Linings, SCL) will further our understanding of stresses around CP openings for different type of linings.

## Conclusions

The current study investigates how PT volume loss and CP shape affect stress redistribution and deformation of the PT lining around the CP opening for shallow tunnels in cohesionless soils. The study utilises 3-D FE analyses and normalised force/moment outputs are extracted from the FE results at springlines and crown/invert of the CP. Volume loss is modelled as a prescribed radial contraction on the parent lining to represent net ground loss during tunnelling. The HSS constitutive model simulated varying soil densities, while various CP/PT size ratios (0.25–0.90), PT volume-loss values (0.25–1.0%) and four CP geometries (circular, square, modified horseshoe, inverted-D) were studied.

Increasing volume loss assigned to the PT lining, (0.25–1.0%) tends to reduce residual hoop forces and hoop bending moments at CP springlines and crowns, but increases longitudinal forces at springlines, especially in loose soils. Thus, volume loss alters both magnitude and location of stress concentrations and must be considered in CP design checks. CP shape strongly influences stress-arching and the redistribution of loads: circular and inverted-D openings concentrate hoop stresses at springlines (effective arching), while square and modified-horseshoe openings produce higher stresses at crown/invert.

Vertical CP deformations decrease with increasing PT volume loss. Square CPs show the largest while circular CPs show the least vertical deformation overall. The relative stiffness of the surrounding ground strongly modifies stress magnitudes and trends. Loose soils amplify, while denser soils reduce the stress around the CP. Designers should therefore take into consideration the shape of the CP especially at higher CP/PT ratios, for effective reinforcement layout.

The normalised force–moment plots developed in this study can be used by tunnel designers to establish the stress regime around a cross passage opening in shallow tunnels constructed in granular soils. The primary input required is a 2D plane-strain analysis of the parent tunnel, from which the hoop force and hoop moment at the springlines are obtained.

These hoop force and moment values can then be used in conjunction with the proposed force–moment plots to estimate the resulting hoop and longitudinal stresses around the CP opening. This will enable the development of an appropriate lining reinforcement strategy in the vicinity of the opening.

## Data Availability

No datasets were generated or analysed during the current study.

## Abbreviations/ Symbols

CP: Cross passage
PT: Parent tunnel
FE: Finite element
FD: Finite difference
CP/PT: Ratio of cross passage diameter to parent tunnel internal diameter
OMC: Optimum moisture content
C/D: Cover-to- diameter (parent tunnel) ratio
CCM: Convergence confinement method
MC: Mohr–Coulomb soil model
HSS: Hardening soil with small strain soil model
t: Thickness of tunnel lining
*w*: Soil water content
*D*_Pr_: Proctor dry density (in %)
*V*_S_: Volume loss associated with parent tunnel lining
*γ*_unsat_: Unit weight of soil in the unsaturated condition (above groundwater table)
*γ*_sat_: Unit weight of soil in the saturated condition (below groundwater table)
*E*_50_^ref^: Reference secant stiffness modulus for primary loading
*E*_oed_^ref^: Reference oedometer stiffness modulus for one-dimensional compression
*E*_ur_^ref^: Reference unloading/reloading stiffness modulus
*G*_o_^ref^: Reference small-strain shear modulus (initial shear stiffness)
m: Exponent controlling stress dependency of stiffness
*γ*_0__.7_: Shear strain at which shear modulus reduces to 70% of its initial value (*G*_0_)
ϕ′: Soil angle of friction
Ψ: Soil dilatancy angle
*K*_0_: Coefficient of earth pressure at rest
*R*_f_: Failure ratio (proximity to failure)
